# The Landscape of Realized Homologous Recombination in Pathogenic Bacteria

**DOI:** 10.1093/molbev/msv237

**Published:** 2015-10-29

**Authors:** Koji Yahara, Xavier Didelot, Keith A. Jolley, Ichizo Kobayashi, Martin C.J. Maiden, Samuel K. Sheppard, Daniel Falush

**Affiliations:** ^1^Biostatistics Center, Kurume University, Kurume, Fukuoka, Japan; ^2^College of Medicine, Institute of Life Science, Swansea University, Swansea, United Kingdom; ^3^Department of Infectious Disease Epidemiology, Imperial College London, London, United Kingdom; ^4^Department of Zoology, University of Oxford, Oxford, United Kingdom; ^5^Department of Medical Genome Sciences, Graduate School of Frontier Sciences, University of Tokyo, Tokyo, Japan

**Keywords:** recombination, selection, pathogenicity, population genomics

## Abstract

Recombination enhances the adaptive potential of organisms by allowing genetic variants to be tested on multiple genomic backgrounds. Its distribution in the genome can provide insight into the evolutionary forces that underlie traits, such as the emergence of pathogenicity. Here, we examined landscapes of realized homologous recombination of 500 genomes from ten bacterial species and found all species have “hot” regions with elevated rates relative to the genome average. We examined the size, gene content, and chromosomal features associated with these regions and the correlations between closely related species. The recombination landscape is variable and evolves rapidly. For example in *Salmonella*, only short regions of around 1 kb in length are hot whereas in the closely related species *Escherichia coli*, some hot regions exceed 100 kb, spanning many genes. Only *Streptococcus pyogenes* shows evidence for the positive correlation between GC content and recombination that has been reported for several eukaryotes. Genes with function related to the cell surface/membrane are often found in recombination hot regions but *E. coli* is the only species where genes annotated as “virulence associated” are consistently hotter. There is also evidence that some genes with “housekeeping” functions tend to be overrepresented in cold regions. For example, ribosomal proteins showed low recombination in all of the species. Among specific genes, transferrin-binding proteins are recombination hot in all three of the species in which they were found, and are subject to interspecies recombination.

## Introduction

Recombination increases the efficacy of natural selection by providing organisms with access to variation that has arisen and been tested in other genetic backgrounds. Within eukaryotes, meiotic recombination is a regular and organized process typically involving a small number of crossovers per chromosome during each meiosis. The rates at which adjacent sites recombine vary by several orders of magnitude along the genome, with hotspots of highly elevated recombination such as programmed double-strand break, or recombination initiation, or gene conversion. The hotspots, and conversely coldspots, have been widely noted in eukaryotes that have been analyzed in detail such as yeast and human (Petes 2001). In human, average length of hotspots and coldspots are estimated to be 19 and 91 kb, respectively (McVean et al. 2004). It has been shown that the location of hotspots in humans can be explained by the binding of the zinc finger protein PRDM9 to specific sequence motifs and that the motif evolves rapidly, leading to high degree of fine scale variability between, and even within, species (Baudat et al. 2010; Myers et al. 2010). At broader chromosomal scales, recombination rates correlate better between species and correlate with factors such as GC content (Fullerton et al. 2001; Meunier and Duret 2004; Duret and Arndt 2008; Marsolier-Kergoat and Yeramian 2009) and the distance to the centromere and telomeres (Marais et al. 2001; Jensen-Seaman et al. 2004; Gaut et al. 2007).

Bacterial chromosomes are typically around 100-fold smaller than those of mammals and the process of recombination is less systematically organized, depending on uptake of naked DNA (transformation), phage infection or conjugation (Dale and Park 2010). Experimental studies have measured variation in transformation frequencies within a bacterial genome (Ray et al. 2009), and the increase in the frequency of transformation associated with sequence features such as DNA uptake sequences (Frye et al. 2013). These studies provide evidence for variation in recombination in the laboratory. Studies that compare multiple genome sequences within a species, analogous to the analyses of recombination in humans that exploit natural variation, have considerable potential for investigating recombination across the genome in natural bacterial populations (Castillo-Ramirez et al. 2012; Joseph et al. 2012; Shapiro et al. 2012; Croucher et al. 2013).

Most commonly, recombination in bacteria involves the replacement of DNA in the recipient genome with homologous sequence from a donor. However, the intensity of this gene-conversion-like process varies markedly between species (Perez-Losada et al. 2006; Vos 2009), ranging from clonal species (Smith et al. 2006) to those that exchange 10% or more of their DNA within a single 4-year human infection (Cao et al. 2014). This variation presents specific challenges for effective comparison between species.

We recently developed a population genetic method for inferring the intensity of recombination at the nucleotide level from large numbers of bacterial genome sequences (Yahara et al. 2014). The approach measures realized recombination which is influenced by patterns of natural selection as well as the rate of genetic exchange. Briefly, our method uses an in silico “chromosome painting” algorithm (Lawson et al. 2012) to identify which strains are most similar in DNA sequence at each part of the genome alignment. The algorithm outputs a matrix indicating which strain is most similar for individual sites and an average across the entire genome. Strains which are recently related through clonal descent will share long stretches of high nucleotide identity and therefore have high average values. Recombination events disrupt genealogical relationships and can lead to high similarity between distantly related strains in the regions that have been transferred. Therefore, in regions of the genome where there have been many recombination events, the local similarity matrix will be more distinct from the genome average than in regions where recombination has been rare. Specifically, we have shown that in simulations of bacterial evolution our measure of difference between average and local similarity values, *D_i_*, is highly correlated with the frequency that sites have been transferred by homologous recombination at that position. The details of the method and its application to simulated and real data sets are provided in a previous publication (Yahara et al. 2014).

The measure applied in this study is for recombination at the population genetic level rather than at direct molecular levels. Furthermore, it is designed to reflect the rate of DNA transfer between cells, rather than the rate at which DNA breakpoints occur at particular sites. Large transfer events affect all the sites in the alignment that are transferred and thus will make a larger impact on our statistics than shorter imports. Our method is not well suited to establishing the particular DNA features responsible for the start and end points of recombination events but instead reflects the various different features that allow DNA to move from cell to cell and to successfully replicate along with the host bacteria.

Here, we investigate the landscape of homologous recombination in pathogenic bacteria by providing the first comparison of variation in intensity of core genome recombination across the chromosome in multiple species. Focusing on diverse collections of ten human pathogens of major public health importance, we analyze alignments of conserved genomic regions of 50 broadly sampled isolates for each species. Our comparative analysis shows that all ten species have hot regions and identifies both common features and differences in the spatial pattern of recombination.

## Results

### Landscapes of Homologous Recombination in Ten Bacterial Species

The 50 isolates in each species were broadly sampled from different clonal groups (as seen in neighbor-joining trees in supplementary fig. S1, Supplementary Material online). The genomes were aligned after eliminating segments that were found in fewer than 40 isolates, for example, transposable elements. We extracted single nucleotide polymorphisms (SNPs) and their positions from the aligned genomic segments, and combined them into genome-wide haplotype data. Statistics regarding the aligned data are shown in supplementary table S1, Supplementary Material online. Average nucleotide diversity in the data ranged from 0.3% (*Neisseria gonorrhoeae*) to 2.2% (*Haemophilus influenzae*). The method that we use to estimate recombination rates is first described in Yahara et al. (2014), with details also provided in Materials and Methods. In the present study, we used a statistic *H_i_* representing recombination hotness or the realized recombination rate at each polymorphic site *i*, by normalizing the statistic *D_i_* (Yahara et al. 2014) that has been shown to be highly correlated with the population genetic parameterρ and the frequency that the site has been transferred by homologous recombination. The normalization enables it to be readily compared between species.

Average and SD of the statistic *D_i_* before the normalization are shown in supplementary table S2, Supplementary Material online. The values are not directly comparable between species because there is a positive relationship between nucleotide diversity and the statistic *D_i_* also seen for other population genetic methods (Yahara et al. 2014). All of the species, however, have distributions of *D_i_* values that are long-tailed and not normal (*P* < 10^−^^15^ by the Kolmogorov–Smirnov testing for normality of the distribution), suggesting presence of hot regions with elevation of the realized recombination rate.

**Fig. 1.**
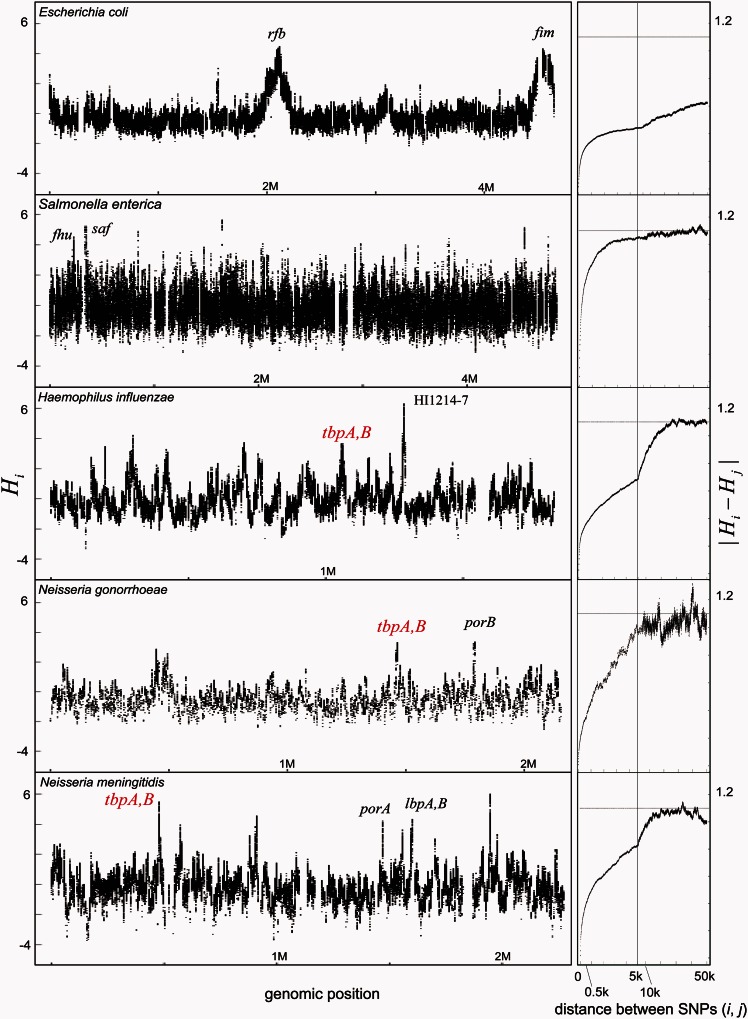

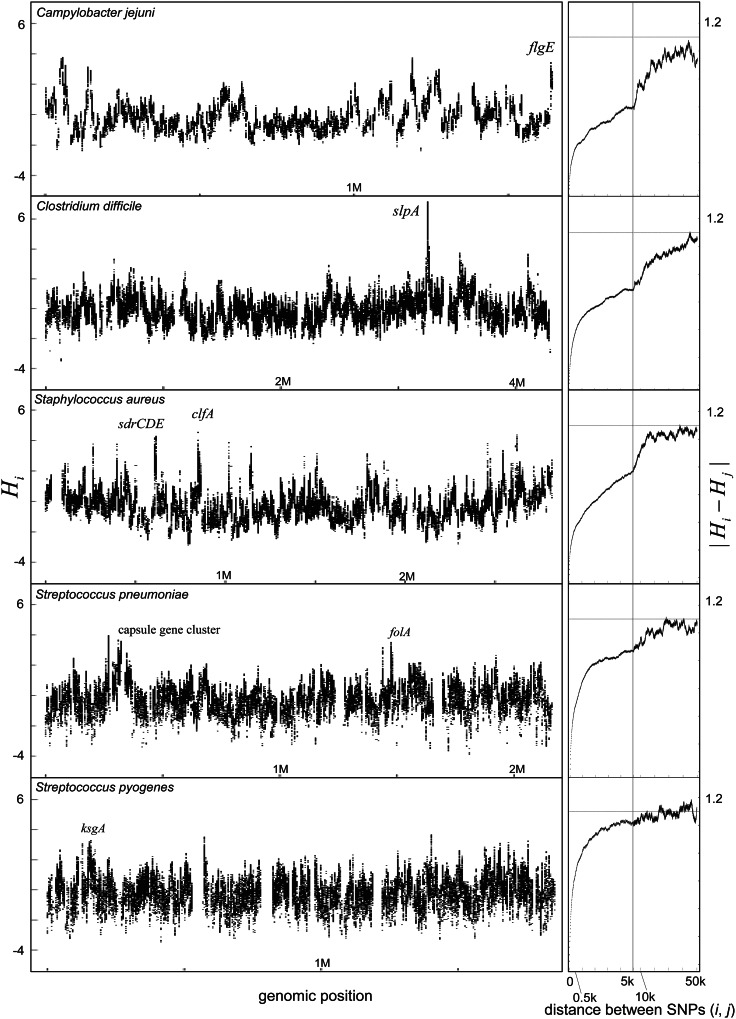
Landscapes of homologous recombination in bacterial species. Left: For each species, values of the per-site statistic (*H_i_*) reflecting relative intensity of recombination at a site (nucleotide) are plotted along the reference genome of each species (supplementary table S1, Supplementary Material online). Some regions devoid of points indicate absence of SNPs for calculation of *H_i_* because the alignment was not obtained in the regions. Locations of some recombination hot regions which are mentioned in the text or table 1 are indicated by letter. Right: Distance-dependence of the per-site statistic is shown in which *x* axis is distance between SNPs (*i, j*) and *y* axis is mean magnitude of the absolute difference of the *H_i_* (normalized *D_i_*) and *H_j_*.

The landscapes of homologous recombination of the ten species inferred by the statistic *H_i_* are shown in figure 1. All species have at least one recombination hot region, defined as having greater than 95% bootstrap support value for the site being in the top two percentiles of *H_i_* values. Genes in the recombination hot regions in each species are shown in table 1 and supplementary table S3, Supplementary Material online. Table 1 lists notable genes that we mention in the main text below or have been mentioned in other studies.

**Table 1.** Notable Recombination Hot Genes.

Species		Recombination Hot Gene	Notes
*Escherichia coli*	Genes in the *rfb* and *fim* gene cluster		Known recombination hot regions
*Salmonella enterica*	*safA*	Outer membrane protein	The fimbrial gene cluster safABCD locating in the centisome 7 genomic island (SCI) (Folkesson et al. 2002); *safA* is implicated in intestinal colonization in swine (Carnell et al. 2007). *safC* is under positive selection (Soyer et al. 2009)
	*safB*	Fimbrial assembly chaperone	
	*safC*	An outer membrane usher protein	
	*fhuA*	Ferrichrome outer membrane transporter	A ferrichrome uptake system *fhuACDB* operon, highly expressed during enteritis (Rollenhagen and Bumann 2006)
	*fhuC*	Iron-hydroxamate transporter ATP-binding subunit	
	*fhuD*	Iron-hydroxamate transporter substrate-binding subunit	
	*fhuB*	Iron-hydroxamate transporter permease subunit	
*Haemophilus influenzae*	*dsbA*	Thiol-disulfide interchange protein	Virulence factor (Rosadini et al. 2008), required for natural transformation (Tomb 1992)
	*tbpA*	Transferrin-binding protein A	See the main text
	*tbpB*	Transferrin-binding protein B	
	HI1217	Haem-utilization protein (hup)	Involving acquisition of heme that is not synthesized in *H. influenza* and thus very important to its survival (Whitby et al. 2009)
	*hxuA*	Heme-hemopexin utilization protein A	
	*hxuB*	Heme-hemopexin utilization protein B	
*Neisseria gonorrhoeae*	*porB*	Major outer membrane protein, PorB	Multiple roles during infection (Chen and Seifert 2013)
	*tbpA*	Transferrin-binding protein A	See the main text
	*tbpB*	Transferrin-binding protein B	
*Neisseria meningitidis*	PL1861	A predicted pilus-associated protein	The highest peak in figure 1, predicted locus PL1861 in GTPS (Kosuge et al. 2006)
	*lbpA*	Lactoferrin-binding protein A	Vaccine candidates as the iron-limitation-inducible outer membrane proteins (Pettersson et al. 2006)
	*lbpB*	Lactoferrin-binding protein B	
	*porA*	Outer membrane protein porin PorA	The two variable regions defining subtypes of this species and eliciting specific bactericidal antibodies in human, which makes it a major component of a number of meningococcal vaccines (Russell et al. 2004)
	*tbpA*	Transferrin-binding protein A	See the main text
	*tbpB*	Transferrin-binding protein B	
*Campylobacter jejuni*	*flgE*	Flagellar hook protein FlgE	A novel recombination hot region which was not reported in Yahara et al. (2014); hypervariability under selective pressure from the host immune system (Luneberg et al. 1998)
*Clostridium difficile*	*slpA*	S-layer proteins on the cell surface	See the main text
*Staphylococcus aureus*	*clfA*	Adhesin	Facilitating bacterial binding to a host (George et al. 2006)
	*sdrC*	Adhesin	Interaction with the extracellular matrix of a host (George et al. 2006)
	*sdrD*		
	*sdrE*		
*Streptococcus pneumoniae*	SP_0346	Capsular polysaccharide biosynthesis protein Cps4A	Capsule biosynthesis gene cluster (Salter et al. 2012)
	SP_0347	Capsular polysaccharide biosynthesis protein Cps4B	
	SP_0348	Capsular polysaccharide biosynthesis protein Cps4C	
	*folA*	Dihydrofolate reductase	Recombination hot (Chewapreecha et al. 2014); drug resistance (Adrian and Klugman 1997)
*Streptococcus pyogenes*	*ksgA*	16S rRNA adenine dimethyltransferase	Its inactivation causes antibiotic resistance (O’Farrell et al. 2004)

In *Escherichia coli*, narrow regions of elevated recombination were detected as well as two broad hot regions (*rfb* and *fim*) greater than 100 kb as previously reported (Touchon et al. 2009; Didelot et al. 2012; Yahara et al. 2014). *Staphylococcus aureus* showed a broad-scale trend toward higher recombination near the origin of replication (position 0) as reported recently (Everitt et al. 2014). In other species, however, there was no evidence of a similar pattern.

To investigate the overall pattern of spatial autocorrelation, we calculated the mean magnitude of the absolute difference of *H_i_* for sites *i* and *j* separated by different physical distances ($\left|H_{i}-H_{j}\right|$). Adjacent sites have values close to zero and in each species the value increases progressively toward the theoretical value expected for unlinked sites (defined in Materials and Methods). In *Salmonella enterica* and *Streptococcus pyogenes*, the value is greater than 0.65 (>60% of the theoretical plateau) after only 500 bp and hot regions are correspondingly very short. In contrast, *E. coli* had the lowest values among the species for distances greater than 10 kb, indicating chromosomal scale variations, nevertheless the values increased rapidly to 0.3 (27% of the theoretical plateau) within the first 500 bp, implying that there is fine scale variation in recombination intensity overlaid onto the broad scale pattern. Even if the two broad recombination hot regions were excluded from the calculation, almost the same result was obtained (supplementary fig. S2, Supplementary Material online). In other species including *Neisseria* spp., *Campylobacter jejuni*, *Haemophilus influenzae, Staphylococcus aureus* and *Clostridium difficile*, the average value of the absolute difference $\left|H_{i}-H_{j}\right|$ increases more steadily with the distance between sites from 0 to 10 kb.

The average value of the absolute difference between sites ($\left|H_{i}-H_{j}\right|$) is similar at each distance between two sites within 2 kb (supplementary fig. S3, Supplementary Material online) whether or not sites *i* and *j* are on the same gene. Therefore, there was no evidence that gene boundaries determine the ends of recombination hot regions.

Each recombination event in the history of our sample affects multiple bases. Part of the measured spatial autocorrelation that we observe therefore reflects the stochastic sampling effects due to the influence of individual events on our statistic, rather than spatial autocorrelation in the underlying distribution of recombination rates. This means that for all the species we are likely in practice to underestimate the true rate at which recombination rates change as a function of position on the genome. However, this sampling effect is probably not in itself the main cause of the differences we see between species, although it may be correlated with it. For example, species where long recombination events are more important will, all else being equal, tend to have true spatial autocorrelation over longer genetic distances and measured autocorrelation over longer due to the proportionately larger effect of the individual recombination events in the history of the sample being studied.

### Comparison between Closely Related Species

**Fig. 2.**
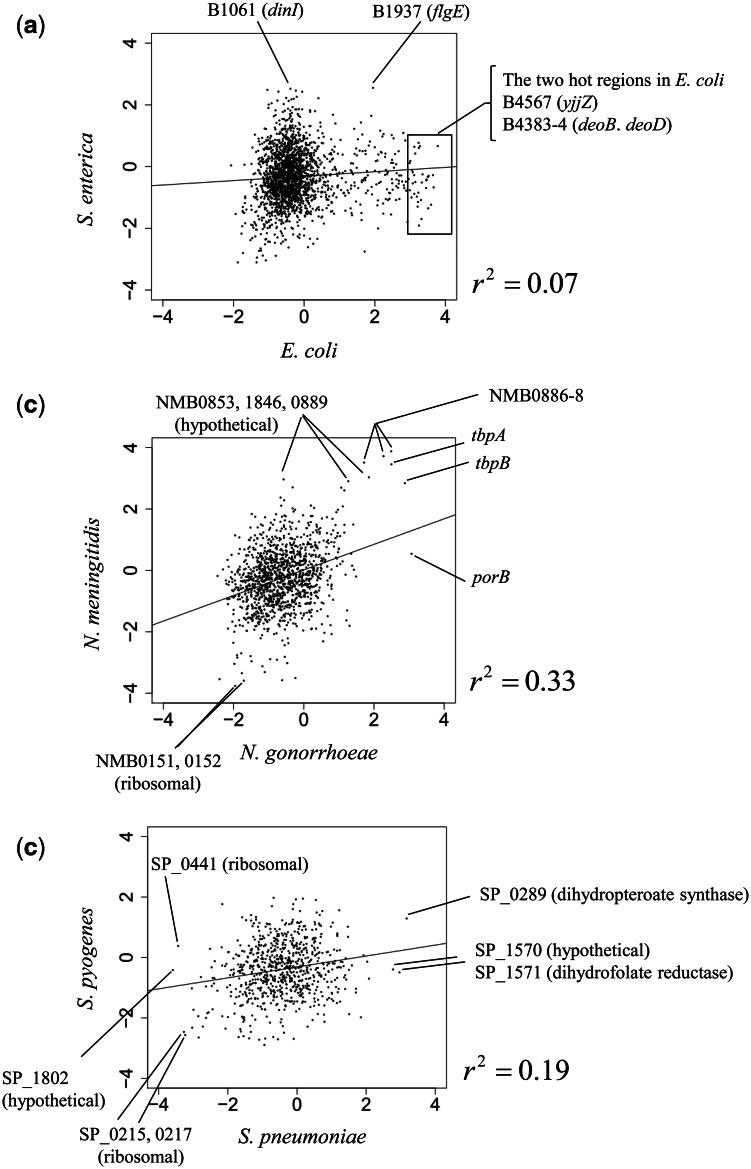
Relation in intensity of recombination between closely related species. Each dot indicates an one-to-one orthologous gene shared between the species. *X* and *Y* axis indicate average values of *H_i_* per orthologous gene in each species.

Among the ten species, there are three pairs of closely related species: *N. gonorrhoeae* and *N. meningitidis*, *E. coli* and *S. enterica*, and *S. pneumoniae* and *S. pyogenes*. Nucleotide divergence between each pair of the species in the core genes shared among the ten species was 5.1%, 13.3%, and 26.8%, respectively. For each of the species pairs, we examined the extent to which the intensity of recombination was correlated among their orthologous genes (fig. 2): *r^2^ = *0.33 between *N. gonorrhoeae* and *N. meningitidis*, *r^2^ = *0.07 between *E. coli* and *S. enterica*, and *r^2^ = *0.19 in *S. pneumoniae* and *S. pyogenes*.

The highest correlation between *N. gonorrhoeae* and *N. meningitidis* is affected by several genes which have high intensity of recombination in both of the species. Among them, *tbpA* (transferrin-binding protein A) and *tbpB* (transferrin-binding protein B) are discussed below. Even when these genes are excluded, however, the correlation is still strong with *r^2^ = *0.28 (*P* < 10^−^^15^). The correlation between *E. coli* and *S. enterica* is lowest, and genes in the two broad hotspots in *E. coli* (31/34 genes with *H_i_* > 0.3) shared no signal of elevated recombination in *S. enterica*.

### Correlation between GC Content and Intensity of Recombination

**Fig. 3.**
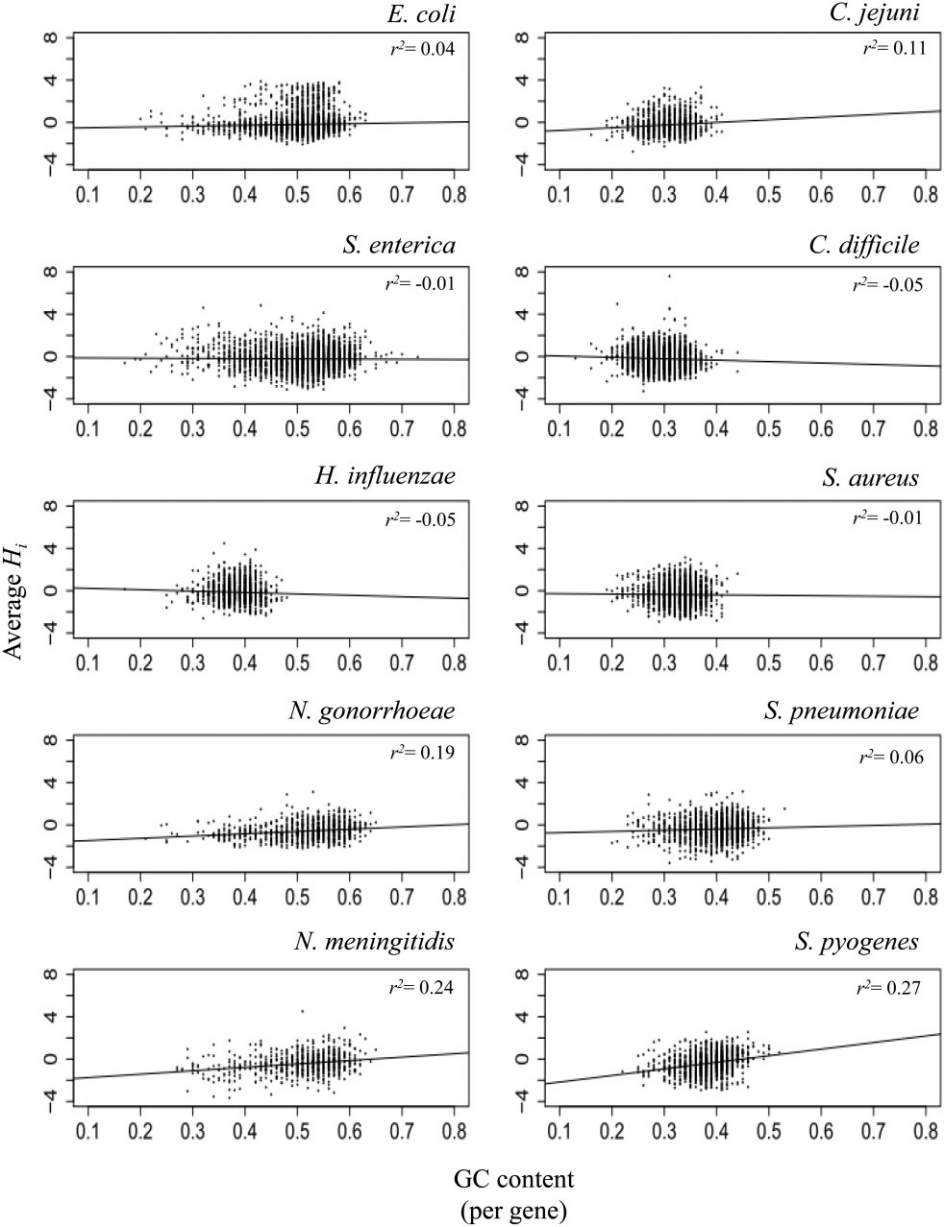
Broad-scale relation between GC content and *H_i_*. Each dot corresponds to a gene. *Y* axis is average *H_i_* per gene. Correlation coefficient (*r^2^*) is indicated.

It is known that local rates of recombination are positively correlated with GC content in several eukaryote genomes (Fullerton et al. 2001; Meunier and Duret 2004; Duret and Arndt 2008; Marsolier-Kergoat and Yeramian 2009), one explanation for which is the GC-biased gene conversion that was experimentally characterized in yeast (Lesecque et al. 2013). Difference in GC content within a bacterial genome has been used to infer horizontally transferred genes (Lawrence and Ochman 1997). We investigated the broad-scale correlation between GC content and average *H_i_* per gene (fig. 3). Among the ten species, strong correlation, comparable to the previous report in humans, was found only in *S. pyogenes*: *r^2^ = *0.26 (*P* < 10^−^^15^). Among the other species, *S. aureus* and *S. enterica* did not show statistically significant correlation, despite considerable variation in GC content in the latter species. Other species showed statistically significant but weak correlation in either direction.

**Fig. 4.**
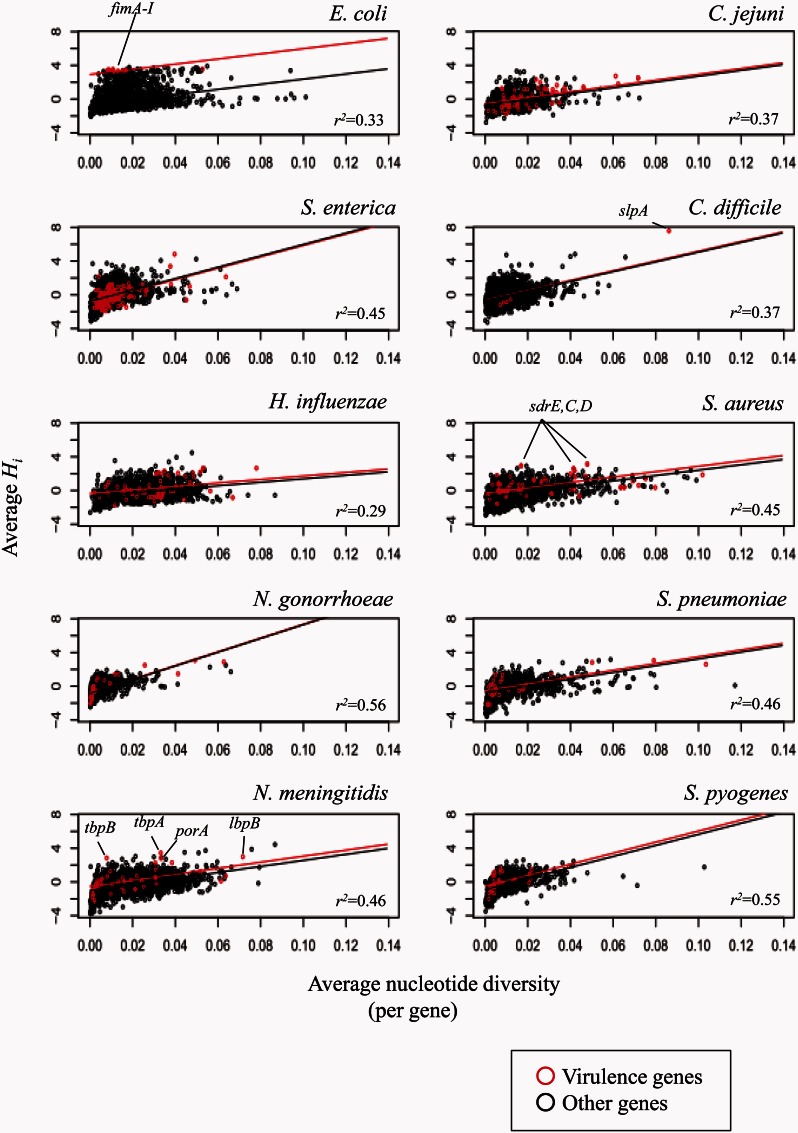
Relationship between average nucleotide diversity and *H_i_* per gene for the virulence genes and other genes in each species. Correlation coefficient (*r^2^*) is indicated. The regression lines compare levels of recombination in the virulence genes and other genes after controlling for the effect of nucleotide diversity.

Nucleotide diversity in some species correlates with GC content (supplementary fig. S4, Supplementary Material online). This covariate might affect the correlations that were observed because the average nucleotide diversity per gene shows a consistent positive correlation with average *H_i_* (fig. 4). After controlling for the effect of nucleotide diversity, the two *Neisseria* species in addition to *S. pyogenes* showed significant positive correlation of GC content on average *H_i_* (*P* < 10^−^^6^) whereas *H. influenzae* showed significant negative correlation (*P* < 10^−^^11^).

Our results can be compared with a recent study that used different statistical methods (Lassalle et al. 2015). Both studies found the strongest positive correlation for *S. pyogenes*, but several species that had strong positive correlations in Lassalle et al. had much weaker correlations in our analysis. One difference may be that their analysis is based on local phylogenetic incongruence, rather than on deviations from clonal descent. Our statistic is therefore likely to put much more weight on long recombination events. Therefore, one interpretation of the discrepancy is that long recombination events have a weaker correlation with GC content than shorter one. Another potential interpretation of the discrepancy between the results of both studies is that phylogenetic incongruence-based methods could be more sensitive than ours to detect recombination events that occurred between recently diverged sequences.

### Recombination Hotness and Virulence

Virulence is often posited to be a key factor in bacterial genome evolution (Hacker et al. 1997). Many important virulence-associated genes are found in hot regions. The highest *H_i_* peak in *S. aureus* spans the SA0742 gene, encoding the important adhesin ClfA, which participates in the infection process by facilitating bacterial binding (George et al. 2006). Another *H_i_* peak was found in the three neighboring genes associated with adhesion (SdrC, SdrD, and SdrE), which may mediate interactions of *S. aureus* with the extracellular matrix (George et al. 2006). This finding may indicate a link between frequent recombination in these adhesion genes in *S. aureus* and bacterial invasion and colonization of the host tissue. Further evidence for a link between frequent recombination in certain genes and bacterial invasion and colonization of the host tissue comes from *S. enterica* where three of four genes in the fimbrial gene cluster *safABCD* were found to be recombination hot. This operon is located in the centisome 7 genomic island (SCI) which contributes to eukaryotic pathogenesis (Folkesson et al. 2002). In *C. difficile,* the single high *H_i_* peak was associated with a cell-wall protein gene cluster containing *slpA* (Dingle et al. 2013).

These examples do not demonstrate systematic differences between virulence genes and the rest of the genome. We therefore took a systematic approach to establish whether the intensity of recombination is elevated in genes that have been annotated as virulence associated. After controlling for the effect of nucleotide diversity, only three species (*E. coli*, *N. meningitidis*, and *S. aureus*) showed significantly higher intensity of recombination in genes annotated as virulence associated (Chen et al. 2012) (supplementary table S4, Supplementary Material online) than other genes at significance level 0.001.

Among the three species, the elevated recombination in virulence-associated loci was most consistent in *E. coli* (fig. 4). The *fim* gene cluster, from *fimA* to *fimI*, is in a recombination hot region. These genes encode the type 1 fimbriae, a crucial factor for virulence during the first steps of infection by mediating adhesion to epithelial cells (Muller et al. 2009).

In *N. meningitidis*, and *S. aureus*, some virulence genes showed elevated recombination. Among them, genes located in the recombination hot regions (table 1) are marked in figure 4. These included *tbpA* and *tbpB—*encoding transferrin-binding proteins, *porA—*encoding the outer membrane protein porin PorA, and *lbpB*—encoding outer membrane lactoferrin-binding proteins in *N. meningitidis* and *sdrC,D,E* encoding three neighboring adhesion genes in *S. aureus*. There were also other virulence genes that did not show the elevation of recombination, indicating that recombination is not necessarily, but can be, associated with virulence—for example, by facilitating bacterial invasion and colonization of the host tissue in the face of a variable immune response.

### Orthologous Genes that Are Recombination Hot in Multiple Species

We found that *H. influenzae*, *N. gonorrhoeae,* and *N. meningitidis* share recombination hot orthologous genes (fig. 1). One of the second highest *Hi* peaks in *H. influenzae* corresponds to two neighboring transferrin-binding protein (*tbp*) genes. One of the highest *H_i_* peaks in *N. gonorrhoeae* corresponds to NGO1495 (*tbpA*, transferrin-binding protein A) and NGO1496 (*tbpB*, transferrin-binding protein B). Furthermore, the second highest peak in *N. meningitidis* also corresponds to the *tbpB* and *tbpA*. The transferrin-binding proteins are outer membrane proteins responsible for iron uptake, and the two *tbp* genes are orthologs shared among *N. gonorrhoeae, N. meningitidis*, and *H. influenzae*. Preferential recombination of DNA fragments spanning the *tbpB* has previously been reported in *N. meningitidis* (Linz et al. 2000) that was attributed to the pressure of the human immune response. Here, we found that the *tbpB* and *tbpA* were recombination hot in all of the three species sharing these orthologous genes. Based on our definition of hot genes as being in the top two percentiles of *Hi*, the probability of observing the shared feature among the three species is the cube of 0.02, which is significant at the level of *P* < 0.05 even after Bonferroni correction by the number of orthologous genes shared among the three species. Allowing for multiple comparisons, there are no other orthologous genes which are overrepresented in hot regions in multiple species.

We also found evidence of interspecies recombination in the two *tbp* genes. Phylogenetic trees (supplementary fig. S5, Supplementary Material online) are consistent with the existence of hybrids resulting from recombination between *N. meningitidis* and *N. gonorrhoeae.* The orthologous gene in *H. influenza* was not included in the analysis because of low sequence identity. Interspecies recombination between *N. meningitidis* and *N. gonorrhoeae* has not previously been reported for *tbpA.* However, previous studies have reported interspecies recombination between *N. meningitidis* and nonpathogenic *Neisseria* spp. in the *tbpB* gene, but they did not report interspecies recombination between *N. meningitidis* and pathogenic *N. gonorrhoeae* (Linz et al. 2000; Harrison et al. 2008). *Neisseria gonorrhoeae* is a sexually transmitted species which usually does not share an ecological niche with *N. meningitidis* in human nasopharynx and has been described as a genetically isolated (Bennett et al. 2007; Hanage 2013).

### Functional Categories with High or Low Recombination Rates

The relative intensity of recombination across the species was also compared for different functional gene categories using the median value of *H_i_* in each category. Although the statistic is constructed so that the mean is 0, the *H_i_* values we obtain have a long positive tail (e.g., supplementary fig. S6, Supplementary Material online) and median values are generally negative.

**Fig. 5.**
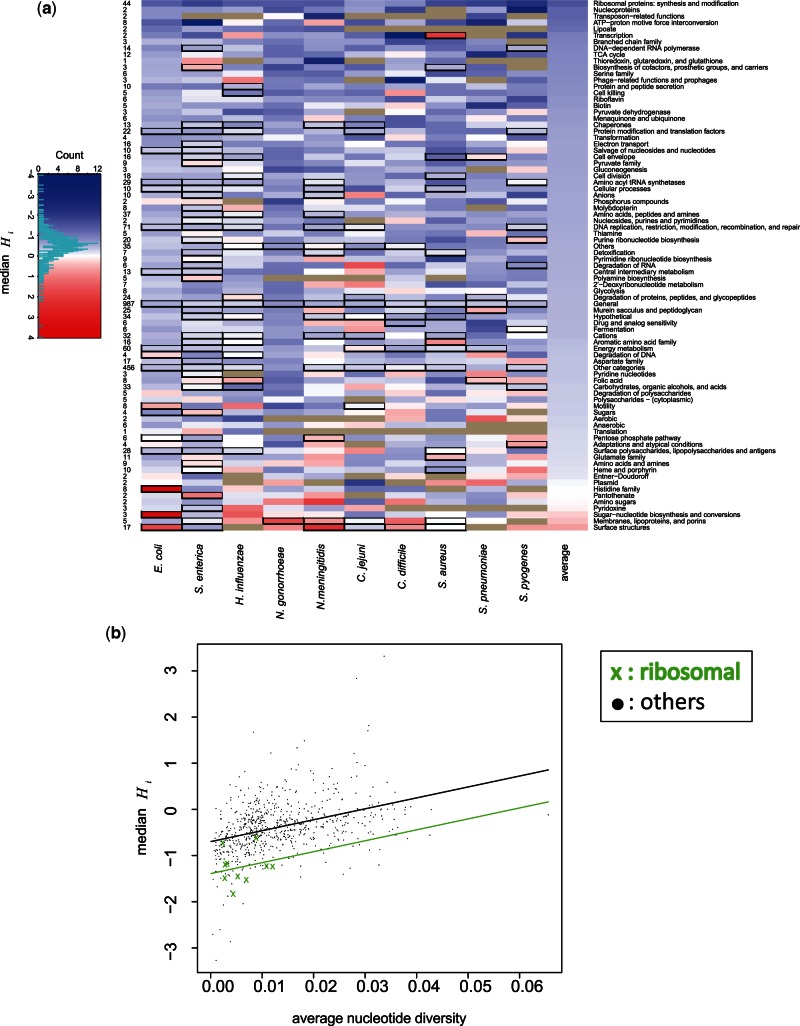
(*a*) Relative intensity of recombination in each functional category in each species. Each cell indicates median of *H_i_*. The rows are sorted by average of the medians of each category across the species (in the most right column). Cells circled by the black rectangles mean presence of a recombination hot gene in the categories. The numbers in the left indicate average number of genes in each category. Gray cells indicate absence of genes in a category of a species. (*b*) Low level of recombination in genes of ribosomal proteins compared with others across the ten species. Each orange x-mark (ribosomal) or black dot (others) corresponds to median of *H_i_* of a functional category of a species in figure 5*a*. The regression lines show low level of recombination in genes of ribosomal proteins after controlling for the effect of nucleotide diversity.

Ribosomal proteins are inferred to have low recombination in all species (fig. 5) but this is influenced by low nucleotide diversity in the genes, as there is a positive correlation between nucleotide diversity and the values of *H_i_* (fig. 4). However, an analysis of covariance showed that the low recombination in the genes of ribosomal proteins remained significant after controlling for the effect of nucleotide diversity (*P* < 10^−^^4^; fig. 5). There are also other gene categories that show low recombination in almost all of the ten species such as nucleoproteins, DNA-dependent RNA polymerase, and TCA cycle which are related to basic cellular functions (fig. 5).

Functional gene categories carrying at least one recombination hot gene in a species are indicated by black rectangles (fig. 5). No category contains hot genes in all species, except “General” which contains genes of diverse functions. The most consistent signal is found across genes categorized as associated with “Surface structures,” “Membranes, lipoproteins, and porins,” and “Cell envelope,” in which nine of the ten species have a recombination hot gene.

## Discussion

Realized recombination analyzed by nucleotide sequence data depends both on the mechanisms that transfer DNA from genome to genome and the selective forces that act on the recombined sequence. Conjugation can lead to transfer of hundreds of thousands of bases from cell to cell in a single event (Dale and Park 2010; Bellanger et al. 2014) but requires the presence of conjugation machinery in the donor cell. Natural transformation can lead to transfer of long DNA fragments up to dozens of kilobases including transposons, integrons, and/or gene cassettes (Domingues et al. 2012). Natural transformation of naked DNA or transfer through phage allows shorter sequence to be incorporated into a cell and furthermore, DNA is often fragmented and partially digested before integration into the recipient genome. Imported DNA is more likely to persist if it confers novel function to a lineage or replaces deleterious mutations, but long imports are also more likely to disrupt existing functional interactions.

Comparative analysis revealed similarities and differences in the chromosomal pattern of recombination of ten human pathogens. The topography of the recombination landscapes differed, even for closely related species. Several species have recombination hot regions that were typically only a few hundred bases, for example, *S. enterica*. Its sister species, *E. coli* has hot regions that are more than 100 kb in length. There are other species, such as *N. meningitidis*, where hot regions are typically a few kilobases in length. Where there is high frequency of imports that span the same set of multiple genes, such as in *E. coli*, it is likely to reflect both opportunities for transfer of large stretches of DNA and the functional interaction of contiguous genes. Such long imports could potentially confer more advantage than short ones, if functional interactions of the imported continuous genes were preserved.

The link between frequent recombination in certain genes and bacterial invasion and colonization of the host tissue was shown in several species; however in *E. coli*, there is also evidence for two very long hot regions. The degree of hotness varies within the long regions, implying that most of the events do not span the whole region. It is nevertheless likely that the recombination events responsible for the signal are substantially longer than those that underlie the short hotspots.

Across the ten species there was no evidence that gene boundaries determine the ends of recombination hot regions. For example, in *N. meningitidis*, *tpbB,* encoding the transferrin-binding protein, is hot due to strong natural selection for new variants during epidemics, but the boundaries of the import events are often several kilobases away from the gene (Linz et al. 2000), leading to a hot region with indistinct edges.

The ten species in this study included three pairs that were relatively closely related, including *E. coli* and *S. enterica*. The other two pairs (from the genera *Neisseria* and *Streptoccocus*) did not show pronounced differences in the lengths of their hot regions. The correlation between orthologous genes was nevertheless relatively weak in the two *Streptococcus* species and moderate between *N. meningitidis* and *N. gonorrhoeae*. Our results therefore demonstrate that recombination hotspots and other quantitative features can change rapidly in bacteria as in mammals (Auton et al. 2012). Such a change might reflect the divergence of the species’ ecological niches and their associated selective pressures.

There was a strong positive correlation between diversity and estimated recombination. As discussed previously (Yahara et al. 2014), this correlation is difficult to interpret in a causal way. Our method can be more sensitive in detecting recombination where diversity is higher. Both realized recombination rates and the rate of substitution are increased by diversifying selection at particular loci. Furthermore recombination can also directly affect the amount of diversity that is observed, for example, by introducing diversity from distant taxa. Finally, sequence diversity can alter the amount of recombination that takes place due to mechanistic factors such as the suppression of recombination between divergent sequences by mismatch repair mechanisms (Tham et al. 2013).

Parts of the mobile accessory genome can have a different GC content than the rest of the genome. However, the effect of GC content has not been related to homologous recombination in bacteria. Individual species did have significant but weak correlations between GC content and recombination but overall, we found no evidence that high or low GC regions are more likely to be transferred between genomes. Specific recombination processes such as transformation might be affected by GC content of the DNA that is being transferred, but these mechanistic factors are not consistent enough between species to leave a systematic signal in our scan. Nor did we find systematic patterns relating to other chromosomal features such as distance to origin of replication.

A feature that was consistent across all the ten species was the relationship to gene function. Ribosomal genes were, on average, recombination cold in every species and were colder than expected based on their low nucleotide diversity. These genes were conserved across species because of their central role in cellular replication and are assumed to be principally under purifying selection. Genes encoding other cellular housekeeping functions were also cold. These genes can be contrasted with the categories of genes encoding surface structures, membranes, lipoproteins and porins, and the cell envelope, all of which involve the interaction of the cell with its environment and each of which was recombination hot. This interaction has often been presumed to lead to species specific and fluctuating selection pressures. Our findings suggest that the contrast between housekeeping and cell surface function does indeed lead to a substantial difference in realized recombination rates.

Most human pathogens have emerged from harmless commensal organisms. The rapid evolution of virulence traits may be facilitated by high rates of recombination, in which case virulence genes would be expected to give a recombination hot signal. This was observed in *E. coli*; however, no consistent signal was observed across the ten species. One explanation for this is that annotation of virulence genes is not adequate, or virulence is a composite term incorporating transmission efficacy, toxin production, and other factors associated with pathogenic human infection. The genes associated with these traits may evolve in very different ways. In other words, the signature of higher realized recombination rate can be useful to detect various traits under positive selection. Using techniques, such as that employed here, it will be possible to investigate broad patterns in the localization of recombination and the mechanistic and selective features involved.

## Materials and Methods

### Isolate Genomes

We obtained data for ten species for which hundreds or thousands of genomes (complete or incomplete) were available in the rMLST (Jolley et al. 2012) BIGSdb (Jolley and Maiden 2010) database. A total of 50 genomes (including a reference complete genome) were used for each species. Isolates were manually chosen from neighbor-joining trees using the rMLST loci (Jolley et al. 2012) of up to 1,000 genomes for each species, including several strains from each major clade, to reflect deep branching population structure. Reference genomes used in this study were as follows: *E. coli* K-12 MG1655 (Blattner et al. 1997), *S. enterica* Typhimurium LT2 (McClelland et al. 2001), *H. influenzae* Rd KW20 (Fleischmann et al. 1995), *N. gonorrhoeae* FA 1090, *N. meningitidis* MC58 (Tettelin et al. 2000), *C. jejuni* NCTC 11168 (Parkhill et al. 2000), *C. difficile* strain 630 (Sebaihia et al. 2006), *S. aureus* N315 (Kuroda et al. 2001), *S. pneumoniae* TIGR4 (Tettelin et al. 2001), and *S. pyogenes* SF370 (Ferretti et al. 2001). The genome sequence data will be available in the Dryad data repository, http://datadryad.org (last accessed November 1, 2015).

### Genome-Alignment, SNP Calling, and Preparation of Genome-Wide Haplotype Data

For each species, contiguous sequences of the 50 genomes were exported from the database. Sequences were aligned using Mugsy (Angiuoli and Salzberg 2011) to construct locally collinear blocks (LCBs). We used all LCBs in which at least 80% (40/50) sequences were aligned. The average number of LCBs satisfying the criterion was 2,176 (ranging from 866 to 4,189) in the ten species. For each of the filtered LCBs, the sequence was extracted from the reference strains, and compared with the reference genome using Basic Local Alignment Search Tool (BLAST) to identify the genome position. SNP calling was conducted, and combined with each LCB, while preserving information of SNP positions, to prepare genome-wide haplotype data. Imputation of polymorphic sites with a missing frequency < = 10% was conducted with a missing frequency ≤10% using BEAGLE (Browning BL and Browning SR 2009), as in a previous study (Yahara et al. 2014). The number of polymorphic sites in each species after imputation was as follows: 298,951 in *E. coli*, 190,858 in *S. enterica*, 156,894 in *H. influenzae*, 26,177 in *N. gonorrhoeae*, 135,999 in *N. meningitidis*, 63,402 in *C. jejuni*, 161,878 in *C. difficile*, 117,564 in *S. aureus*, 87,800 in *S. pneumonia*, and 70,064 in *S. pyogenes*. The data were used to construct neighbor-joining trees of each species by MEGA6 (Tamura et al. 2013).

### Inference of Landscapes Hot Regions of Homologous Recombination

“orderedPainting” (Yahara et al. 2014) was applied for the genome-wide haplotype data of each species to infer landscapes of homologous recombination using the per-site statistic (*D_i_*) as a measure of relative intensity of recombination at a site (nucleotide) *i*. It is based on the chromosome painting algorithm (Lawson et al. 2012) that regards a single haplotype on the chromosome of a “recipient” individual as a mosaic, and reconstructs it using haplotypes from all other individuals as potential donors by estimating posterior probability of donors for each polymorphic site on a recipient genome. The chromosome painting thus infers the most recent recombination along a recipient genome and discards a large amount of information in the presence of clones because the clonally related recipient isolates will be inferred to receive almost all of their genome from their clones. To solve these problems and infer intensity of recombination along a recipient genome, orderedPainting conducts the chromosome painting by randomly ordering haplotypes. Namely, for each ordering *j*, it conducts the chromosome painting by conditioning donors of each recipient haplotype (*H_2_*,…… *H_n_*) on the previous ones such that

$$\begin{array}{l} H_{2}|H_{1},\\ H_{3}|H_{2},H_{1},\\ H_{4}|H_{3},H_{2},H_{1},\\ ...\\ H_{n}|H_{n-1},...,H_{1}, \end{array}$$

where (*H_1_*,*H_2_*, . . .…, *H_n_*) is the ordered sample of *n* haplotypes. The estimated posterior probability of donors for each polymorphic site on a recipient genome can be formatted as a matrix in which rows represent recipients and columns represent donors, with the values being normalized so that each row sums up to 1, which is called as the site-by-site copying probability matrix $S_{i}{}_{j}$ of site *i* and ordering *j*. By taking the average of the site-by-site copying probability matrix $S_{i}{}_{j}$for all sites, an average copying probability matrix $A_{j}$ is calculated for ordering *j*. For each site *i* and each ordering *j*, it calculates *d_ij_* as the sum of squared distance of every element of $S_{i}{}_{j}$and $A_{j}$: $d_{ij}=\sum_{}{\left(S_{i}{}_{j}-A_{j}\right)}^{2}$. Then, it calculates the per-site distance statistic *D_i_* by taking the summation of *d_ij_* across orderings as $D_{i}=\sum_{j}d_{ij}$. The statistic captures the extent of deviation of a specific site compared with the genome-wide average, and is highly correlated with local recombination rate (Yahara et al. 2014). Hot regions were defined as sites within the top two percentiles of the distribution of the statistic, and genes with these regions were considered recombination hot. Gene information was taken from reference genomes in MBGD (Uchiyama 2003).

We normalized values of *D_i_* of each species so that their mean and SD became 0 and 1, respectively, in each species. We designated the normalized statistic as *H_i_* and used it for comparative analyses among the species. Distance-dependence of the statistic in each species was examined by calculating absolute value of difference of the normalized statistic between pairs of SNPs *i* and *j* ($\left|H_{i}-H_{j}\right|$) within 50 kb, and plotting its average against distance between the SNPs. Since *H_i_* is normalized, its values are distributed as Normal (0,1) and the difference in absolute value between two such independent and identically distributed values is on average approximately 1.1 as can be computed, for example, using the following R command “mean(abs(rnorm(1e6)-rnorm(1e6))).”

### Ortholog Clustering, and Analysis of Correlation of Recombination Hotness between Closely Related Species

Ortholog clustering was conducted using the reference genomes and DomClust (Uchiyama 2006) implemented in RECOG (http://mbgd.genome.ad.jp/RECOG/). It assigned a functional category in MBGD (Uchiyama 2003) to each orthologous gene. When multiple domains were found in an orthologous gene, multiple functional categories were assigned to it. For the three pairs of closely related species (*E. coli* and *S. enterica*, *N. gonorrhoeae* and *N. meningitidis*, and *S. pneumoniae* and *S. pyogenes*), we calculated average *H_i_* of each one-to-one orthologous gene and examined their correlation by the linear regression.

### Analysis of Relationship between GC Content and Recombination Hotness

GC content was calculated for each gene in a reference genome by EMBOSS (Rice et al. 2000). We also calculated average *H_i_* for each gene and examined correlation between them. We calculated the Pearson’s correlation coefficient. We also conducted multiple linear regression to test the effect of GC content after controlling for the effect of nucleotide diversity: $y_{j}=\beta_{0}+\beta_{1}x_{1,j}+\beta_{2}x_{2,j}+\epsilon_{j}$ where, for gene *j* in a species, *y_j_* is average *H_i_*; *x*_1_*_,__j_* is GC content; *x*_2_*_,__j_* is the average nucleotide diversity; $\beta_{0}$is intercept; $\beta_{1}$ and $\beta_{2}$ are regression coefficients; and $\epsilon_{j}$ is error, which is normally distributed.

### Analyses of Relationships between Nucleotide Diversity, Recombination Hotness, and Virulence

Average nucleotide diversity was calculated for each gene in each species using a sliding window implemented in VariScan version 2.0 (Hutter et al. 2006). The average was calculated from the values of per-site nucleotide diversity for each gene. We calculated the Pearson’s correlation coefficient between the average nucleotide diversity and average *H_i_* per gene.

Analysis of covariance was conducted to test the difference between virulence genes and others in a species in terms of intensity of recombination after controlling for the effect of nucleotide diversity: $y_{j}=\beta_{0}+\beta_{1}x_{1,j}+\beta_{2}x_{2,j}+\epsilon_{j}$ where, for gene *j* in a species, *y_j_* is average *H_i_*; *x*_1_*_,__j_* is a dummy variable distinguishing virulence genes and others; *x*_2_*_,__j_* is the average nucleotide diversity*;* $\beta_{0}$is intercept; $\beta_{1}$ and $\beta_{2}$ are regression coefficients; and $\epsilon_{j}$ is error, which is normally distributed. The virulence genes were defined according to the Virulence Factor Database (VFDB) (Chen et al. 2005, 2012).

### Detection of Interspecies Recombination in tbpA and tbpB Genes

We obtained nucleotide sequences of orthologs of *tbpA* and *tbpB* by BLAST against the genomic contigs of *N. gonorrhoeae* and *N. meningitidis*. Gene presence was defined as a BLAST match of greater than 70% identity and greater than 50% of the locus length (Meric et al. 2014). We also tried to include *H. influenzae*, but the criterion was not satisfied because of larger sequence divergence. The nucleotide sequences of the orthologous genes were aligned by MAFFT (Katoh and Toh 2008). Neighbor-joining trees were constructed by using MEGA 6 (Tamura et al. 2013).

### Analyses of Functional Gene Categories

For each functional gene category in each species, the median *H_i_* was calculated to explore functional gene categories with consistently high or low recombination across the species. These were visualized as a heatmap. Average nucleotide diversity was also calculated for each category in each species. This was based on calculation of nucleotide diversity at a polymorphic site using sliding windows implemented in VariScan version 2.0 (Hutter et al. 2006) to the genome-wide haplotype data. The average was calculated from the values of per-site nucleotide diversity for each gene category. We then conducted analysis of covariance to test the difference between genes of ribosomal proteins and others in terms of intensity of recombination after controlling for the effect of nucleotide diversity: $y_{j}=\beta_{0}+\beta_{1}x_{1,j}+\beta_{2}x_{2,j}+\epsilon_{j}$ where, for gene category *j* in a species, *y_j_* is median *H_i_*; *x*_1_*_,__j_* is a dummy variable distinguishing the category of ribosomal proteins and others; *x*_2_*_,__j_* is the average nucleotide diversity; $\beta_{0}$is intercept; $\beta_{1}$ and $\beta_{2}$ are regression coefficients; and $\epsilon_{j}$is error, which is normally distributed.

The ortholog clustering was also utilized to explore orthologous genes which were recombination hot in multiple species. For each of the genes, we calculated probability of observing the shared feature among *m* species as 0.02*^m^* (*m* = 2 or 3 or … 10) given the assumption that the top two percentiles of sites are recombination hot in each species. The probability was multiplied by the number of orthologous genes shared among *m* species to calculate Bonferroni-corrected *P* value.

## Supplementary Material

Supplementary figures S1–S6 and tables S1–S4 are available at *Molecular Biology and Evolution* online (http://www.mbe.oxfordjournals.org/).

Supplementary Data
