# Supplementary material for: The Landscape of Realized Homologous Recombination in Pathogenic Bacteria
Source: Mol Biol Evol. 2015 Oct 29;33(2):456–71. doi: 10.1093/molbev/msv237 (PMC4866539; doi:10.1093/molbev/msv237)
Supplement: Supplementary Data [file supp_33_2_456__index.html]

The Landscape of Realized Homologous Recombination in Pathogenic Bacteria — The Landscape of Realized Homologous Recombination in Pathogenic Bacteria — Supplementary Data 

# The Landscape of Realized Homologous Recombination in Pathogenic Bacteria

## Supplementary Data

files

- Supplementary Data - zip file
