# Supplementary material for: The Landscape of Realized Homologous Recombination in Pathogenic Bacteria
Source: Mol Biol Evol. 2015 Oct 29;33(2):456–71. doi: 10.1093/molbev/msv237 (PMC4866539; doi:10.1093/molbev/msv237)
Supplement: Supplementary Data [file supp_msv237_suppl_data.zip › FiguresS1-6.pdf]

Figure S1. Neighbor-joining tree of the genome core of each species. (a) *E. coli.* (b) *S. enterica*. (c) *H. influenzae*. (d) *N. gonorrhoeae*. (e) *N. meningitidis*. (f) *C. jejuni*. (g) *C. difficile*. (h) *S. aureus*. (i) *S. pneumoniae*. (j) *S. pyogenes*.

Figure S2. Distance dependence of H_i in *E. coli* after excluding the two broad recombination hot regions.

Figure S3. Distance dependence of H_i calculated separately for sites on the same genes and those on different genes. x and y-axis are the same as those of the right part of Figure 1.

Figure S4. Relations between average nucleotide diversity and GC content.

Figure S5. Signals of inter-species recombination in the within-species recombination hot genes. Neighbor-joining trees of the two genes constructed by a procedure in Methods. **(a) *tbpA*. (b) *tbpB*.**

Figure S6. Distribution of H_i in *E. coli*.

a. *E. coli*

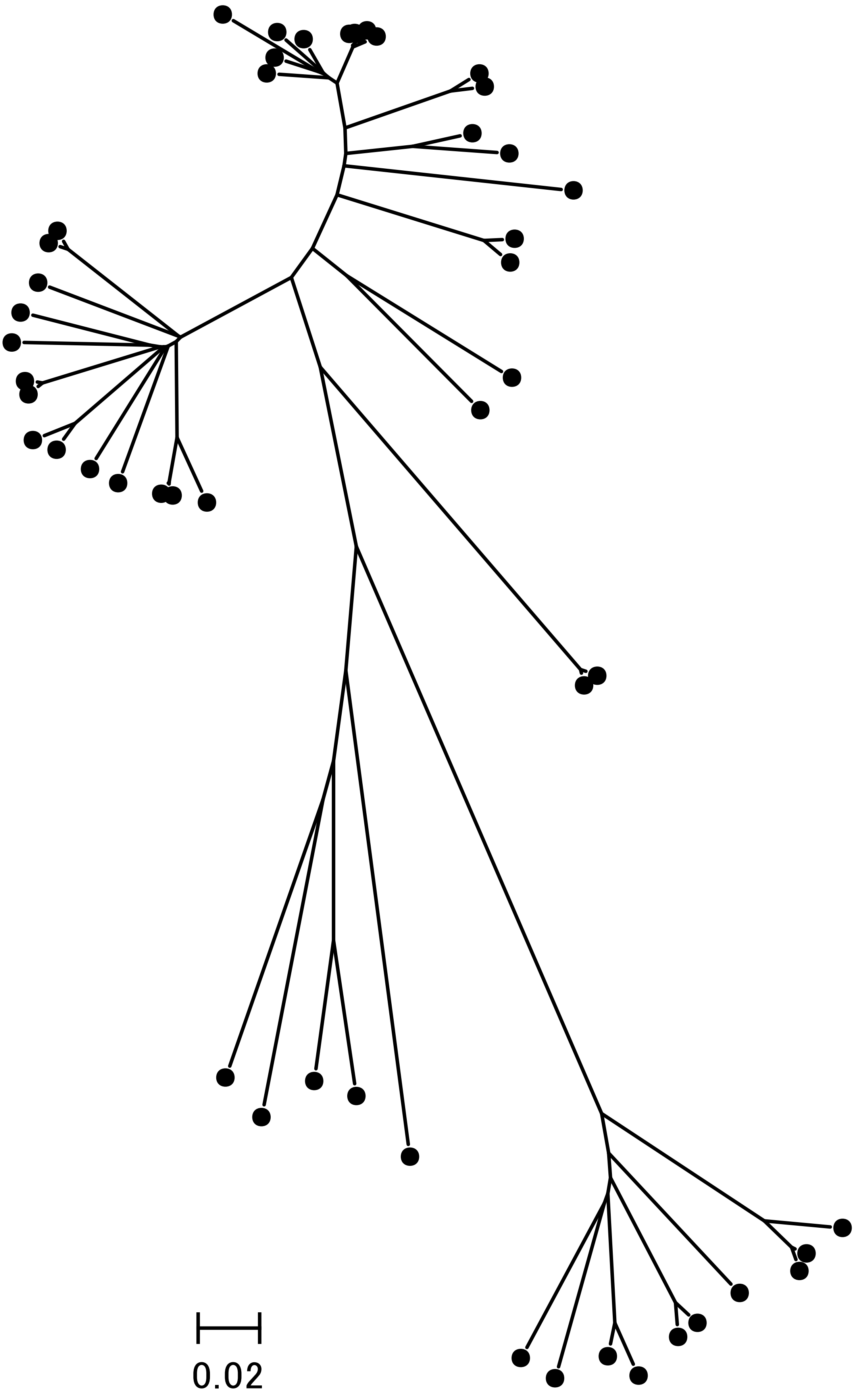

b. *S. enterica*

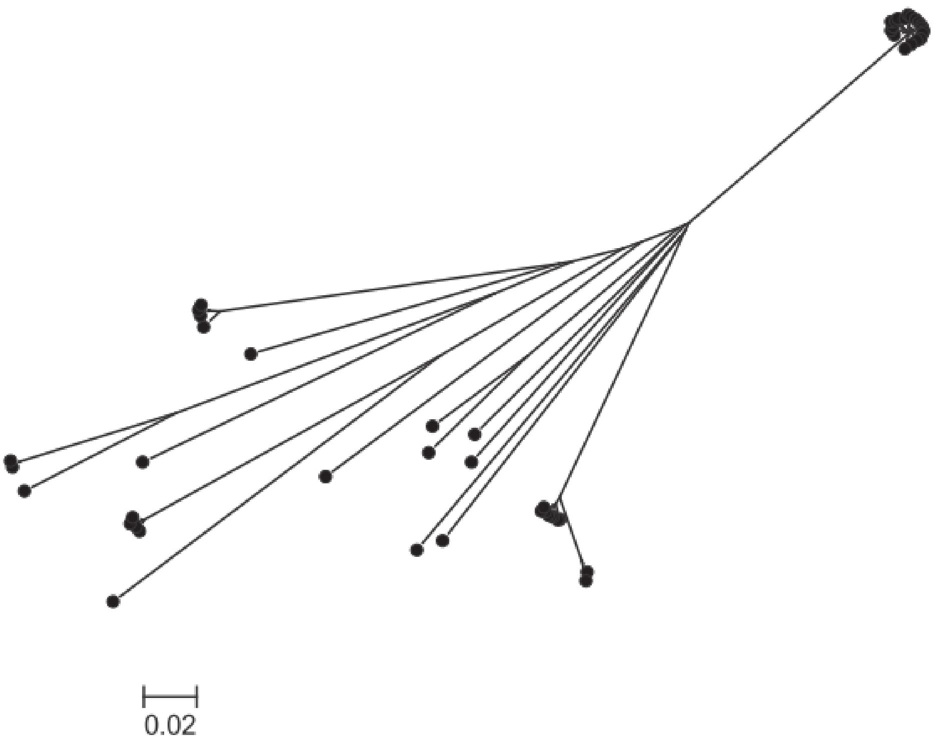

c. *H. influenzae*

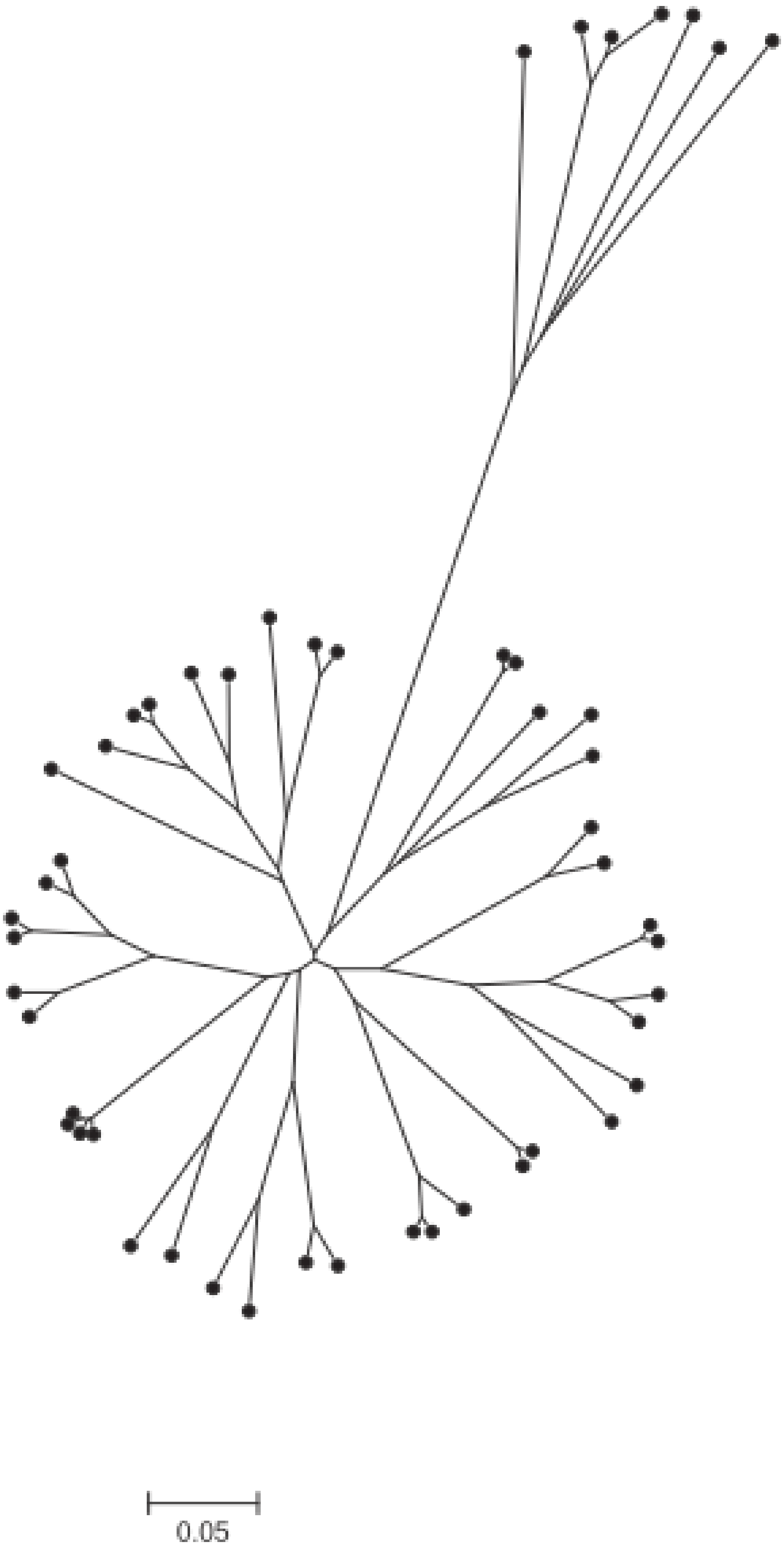

d. *N. gonorrhoeae*

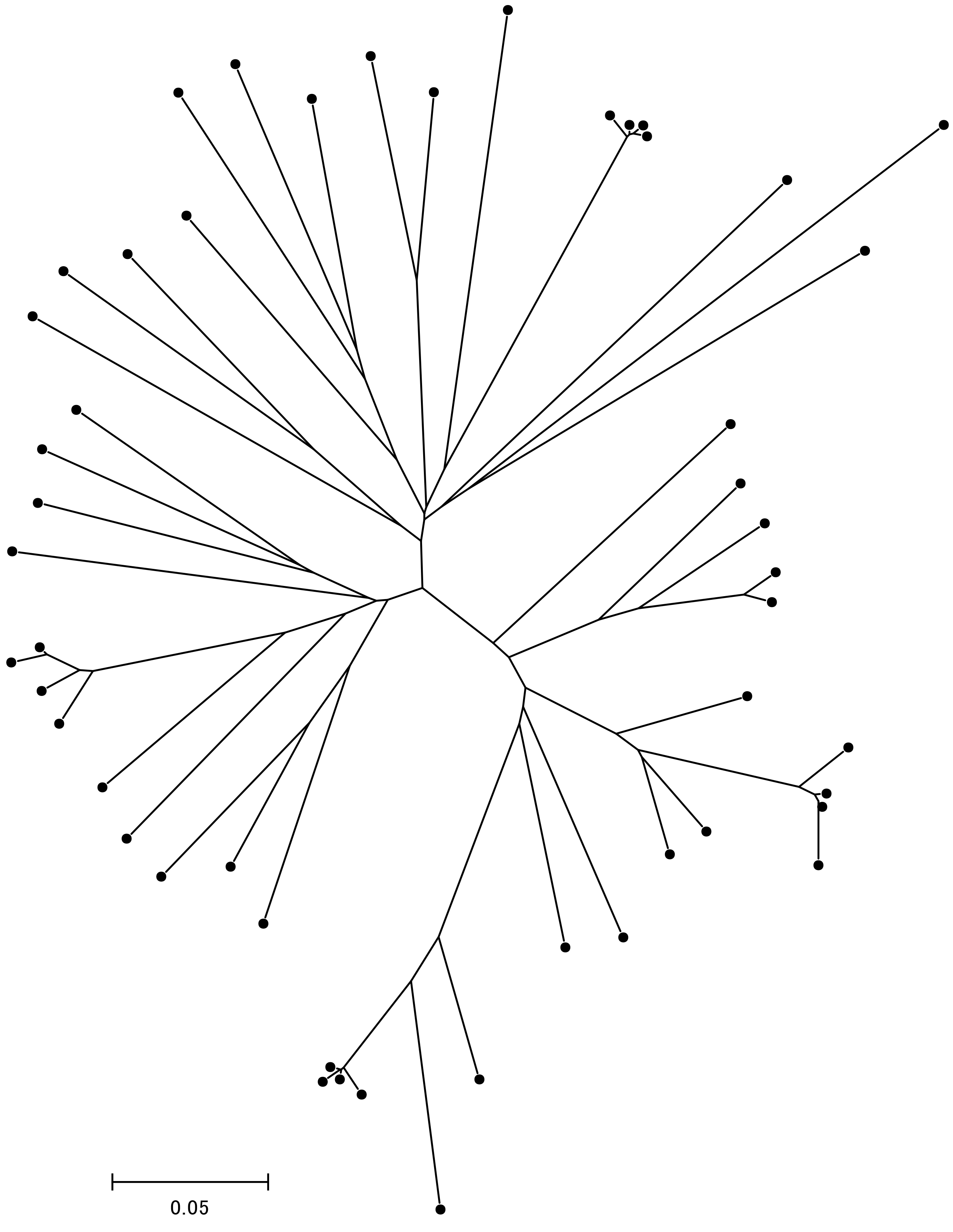

e. *N. meningitidis*

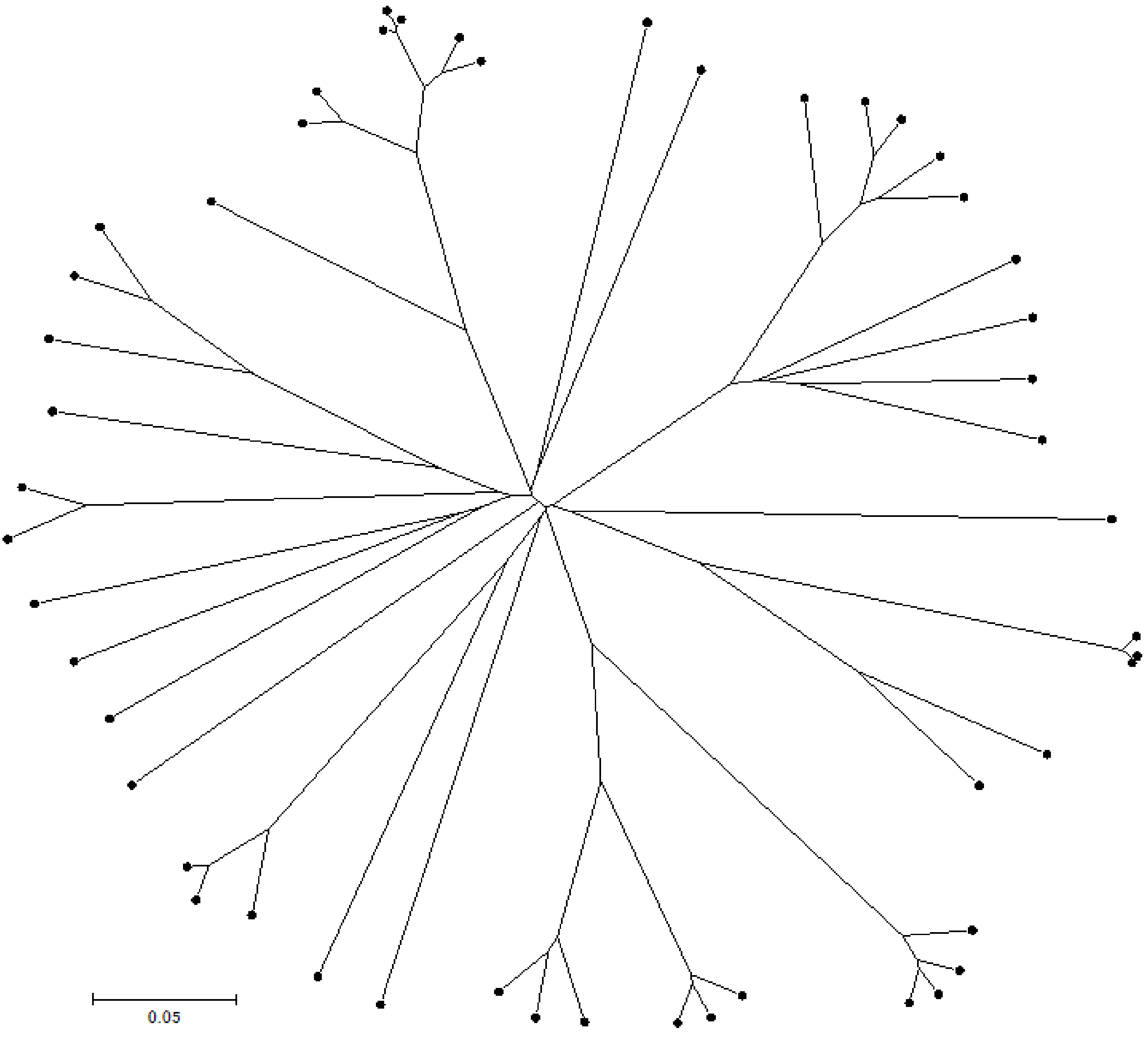

f. *C. jejuni*

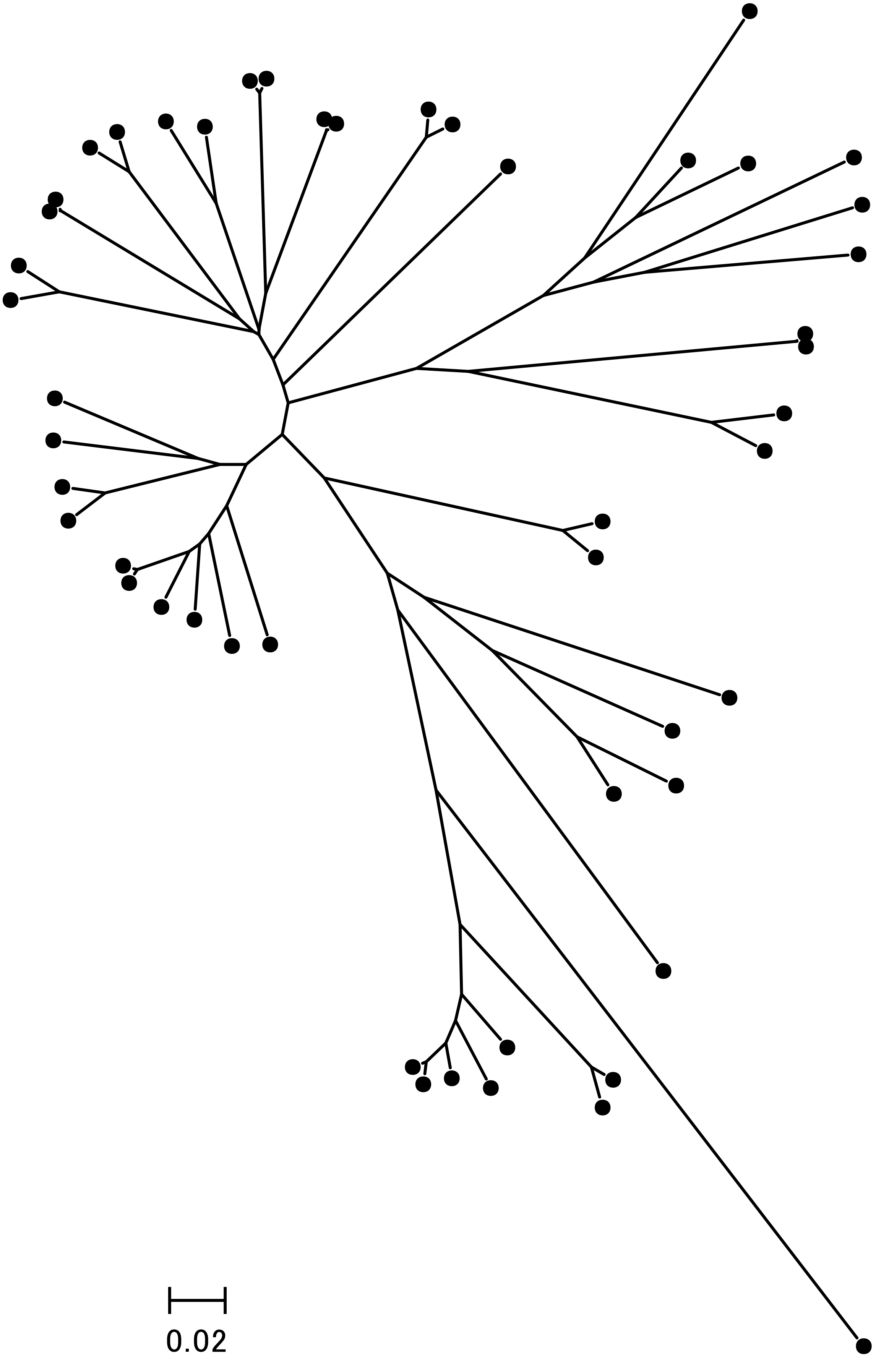

g. *C. difficile*

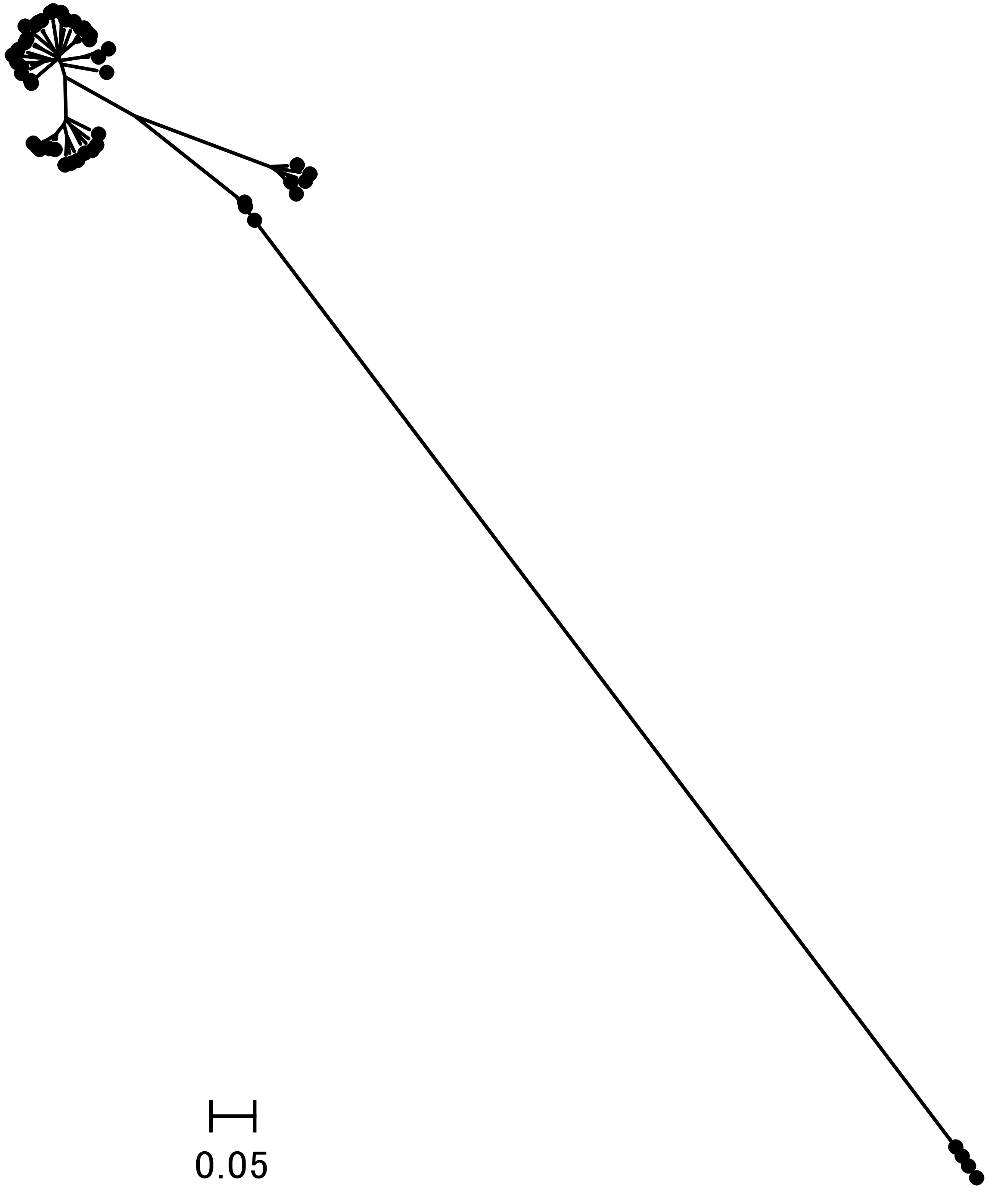

h. *S. aureus*

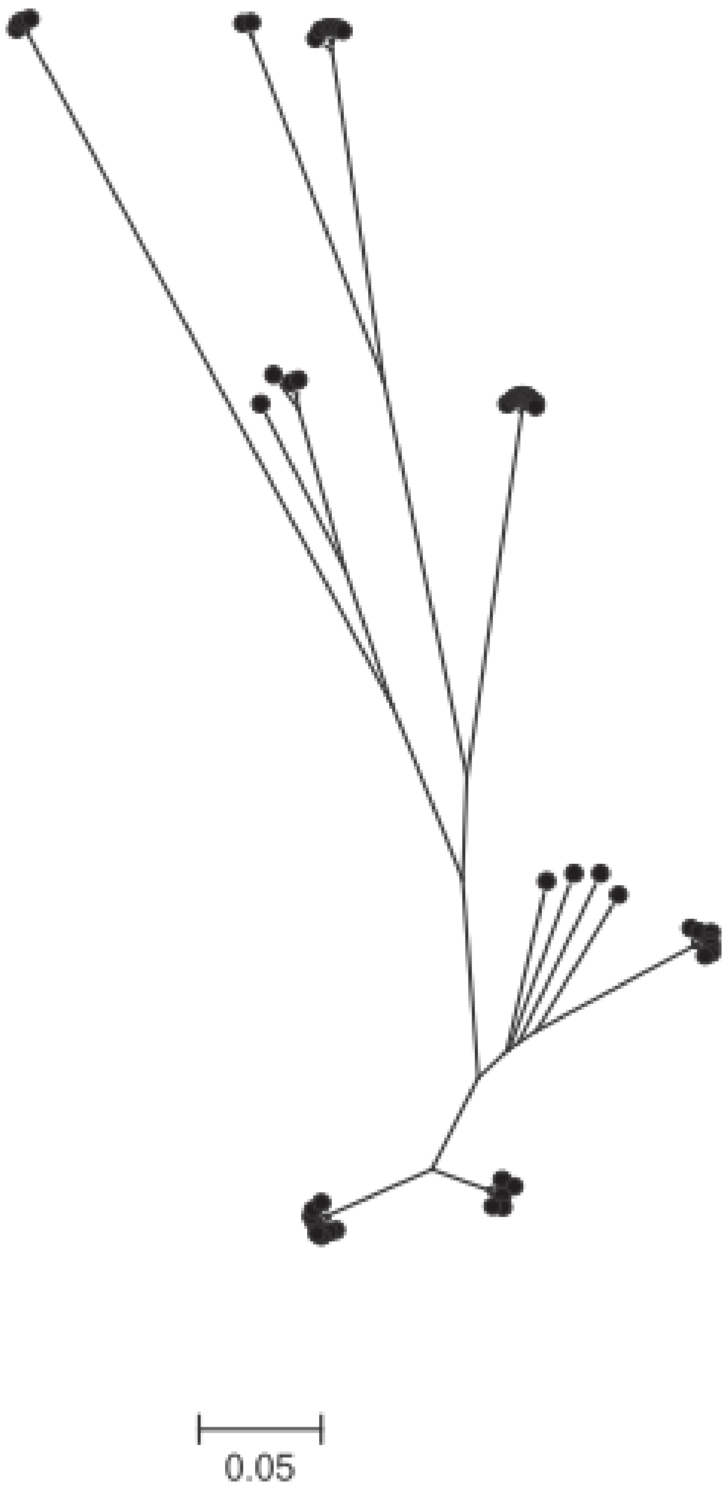

Figure S1

i. *S. pneumoniae*

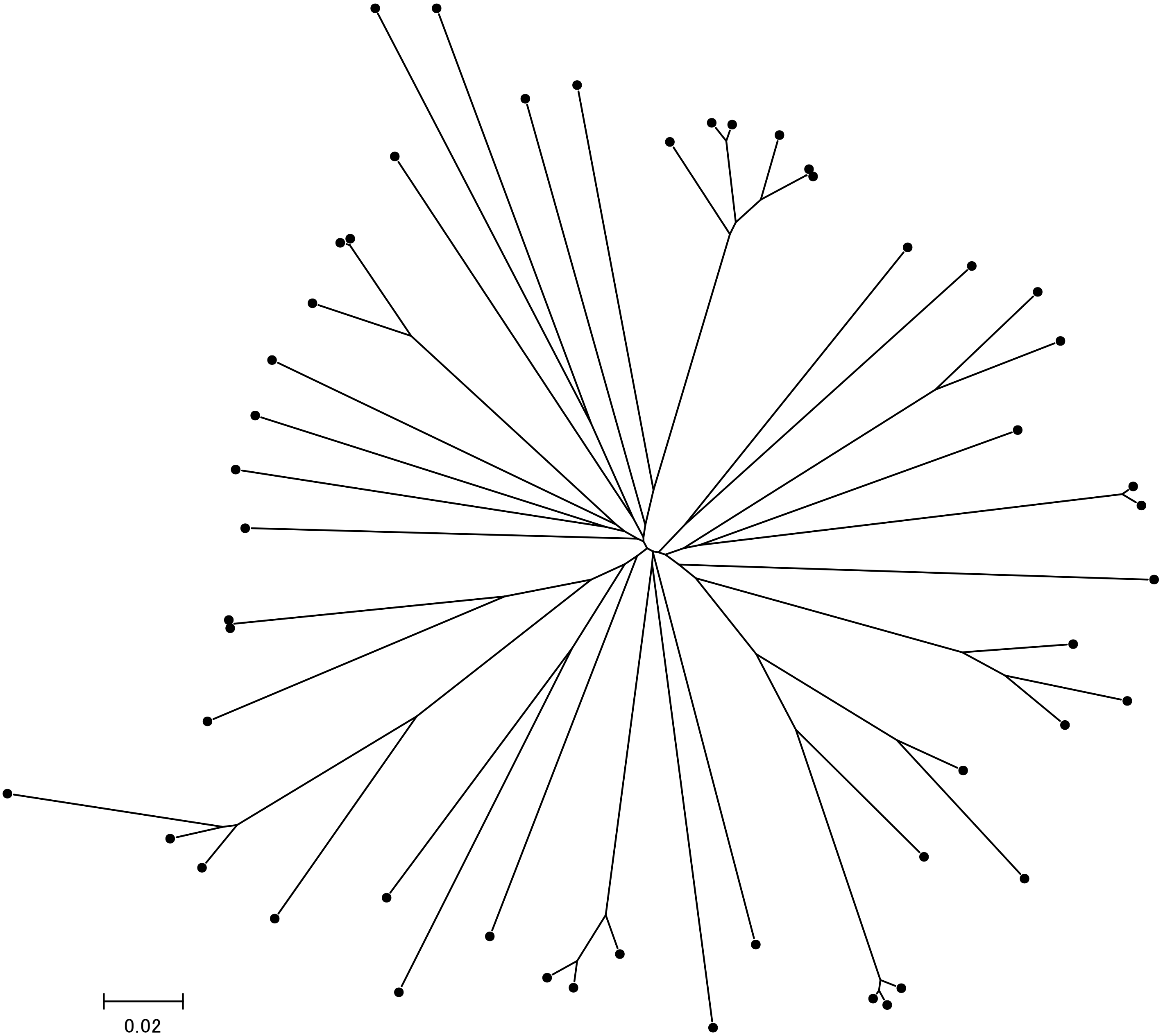

j. *S. pyogenes*

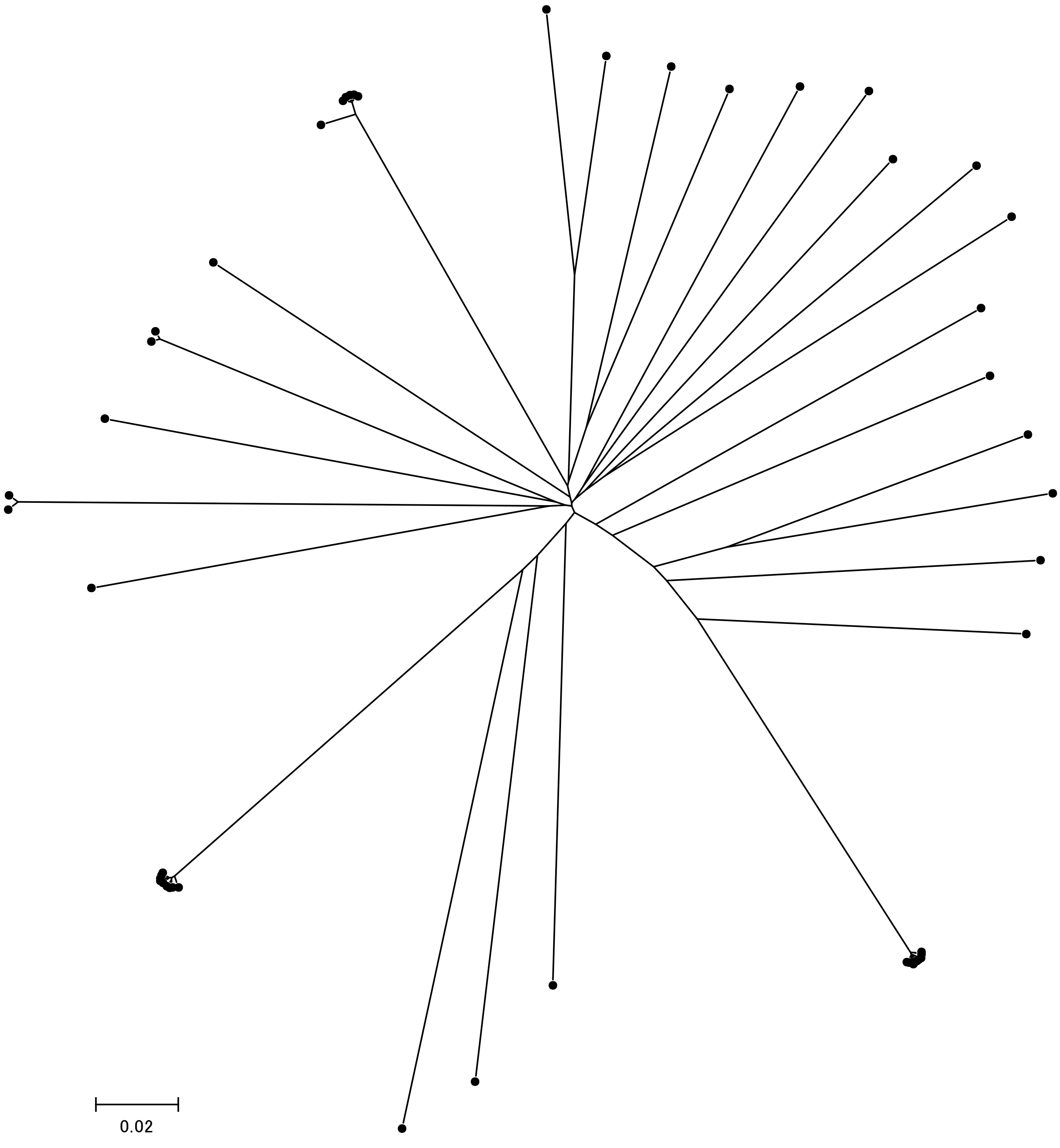

Figure S2

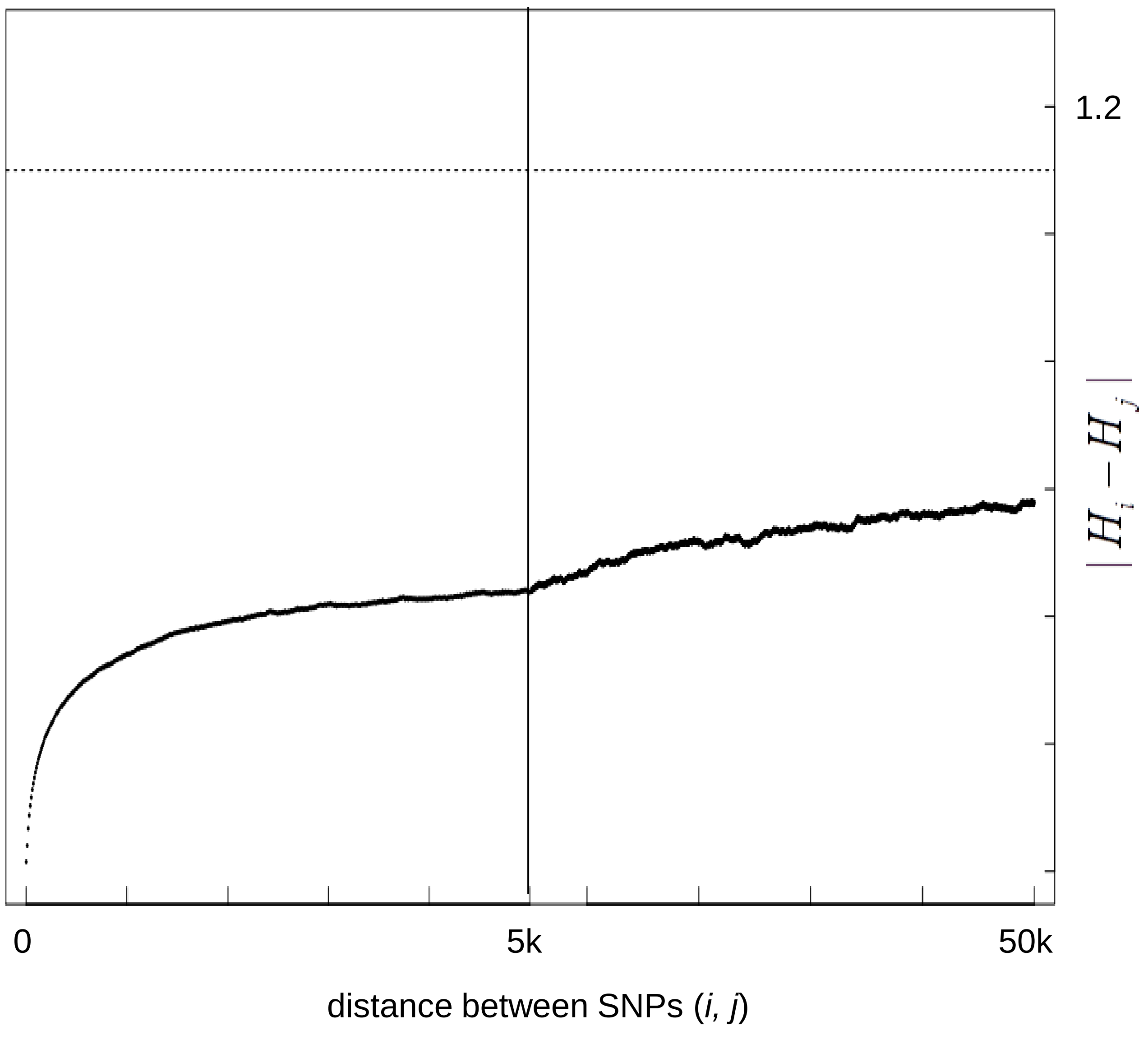

Figure S3

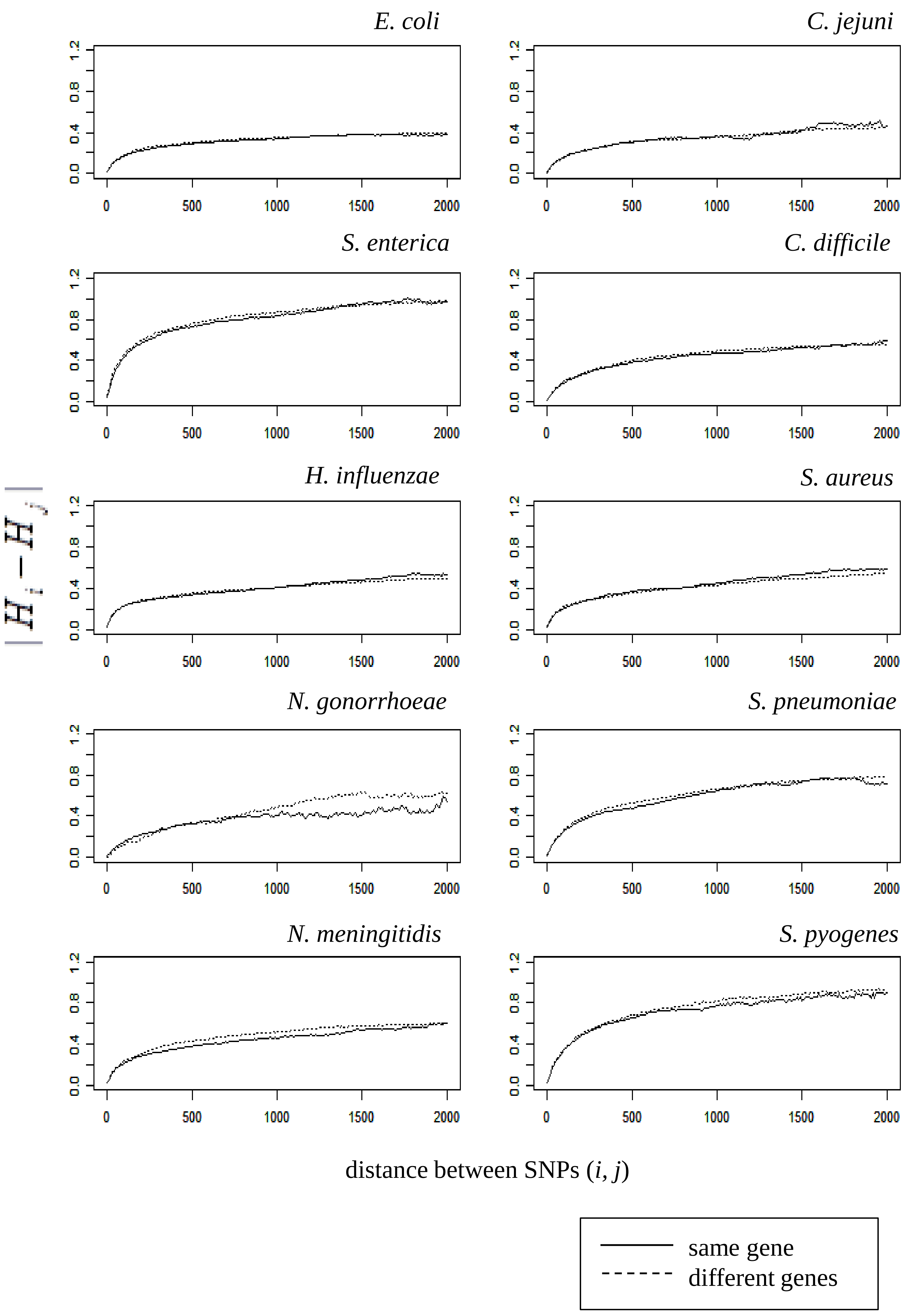

E. coli
C. jejuni
S. enterica
C. difficile
H. influenzae
S. aureus
N. gonorrhoeae
S. pneumoniae
N. meningitidis
S. pyogenes
0.0
0.4
0.8
1.2
0
500
1000
1500
2000
$|H_i - H_j|$
distance between SNPs (i, j)
same gene
different genes

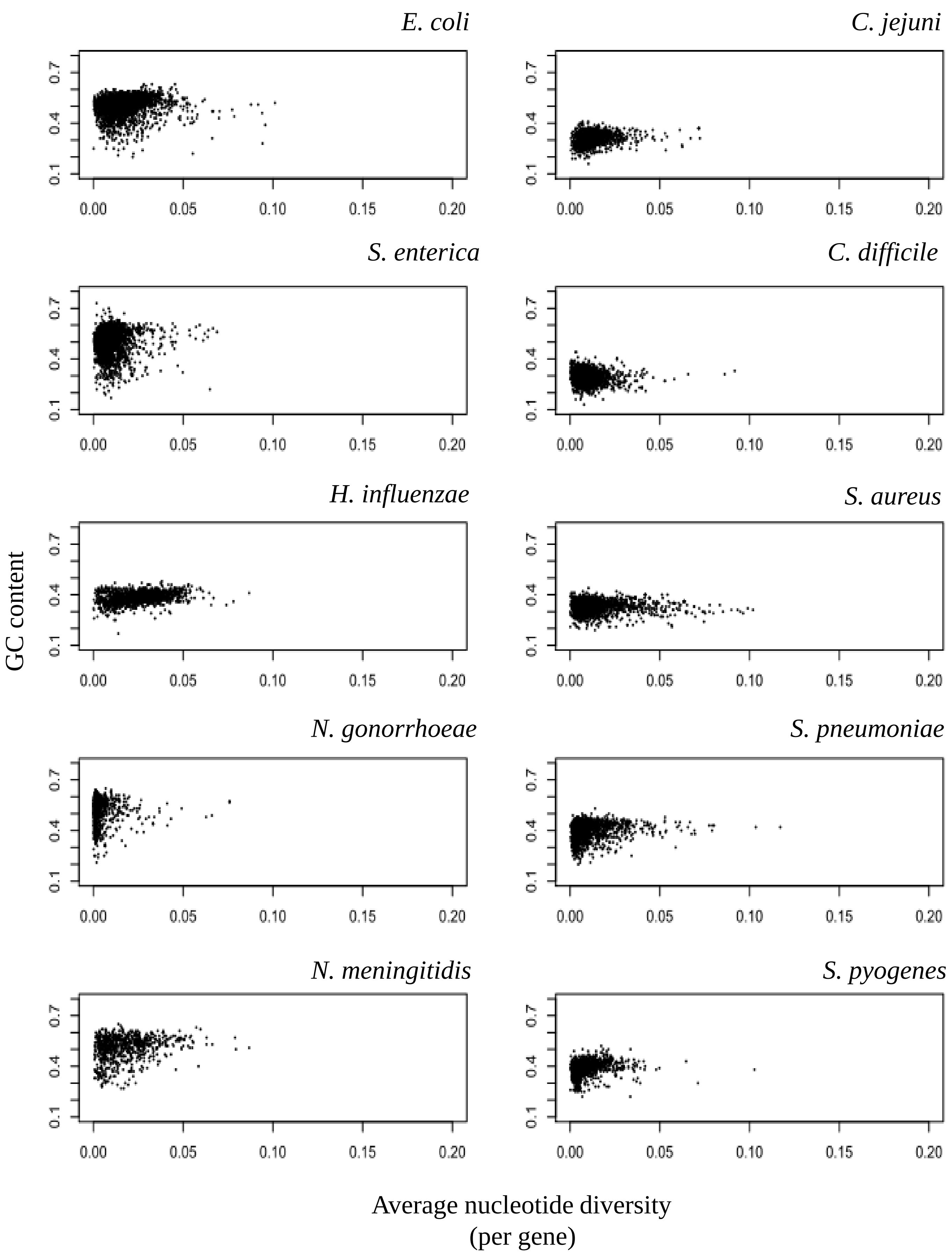
E. coli
C. jejuni
S. enterica
C. difficile
H. influenzae
S. aureus
N. gonorrhoeae
S. pneumoniae
N. meningitidis
S. pyogenes
GC content
0.1
0.4
0.7
0.00
0.05
0.10
0.15
0.20
Average nucleotide diversity
(per gene)

Figure S5

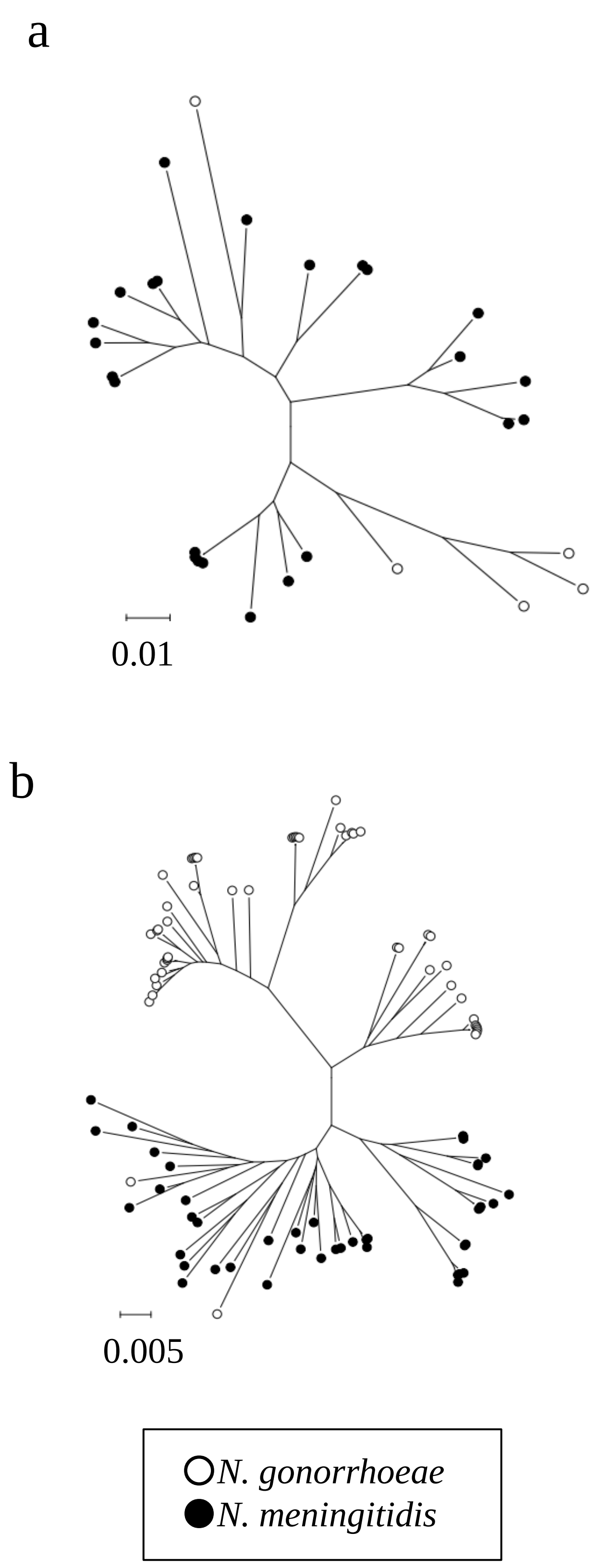

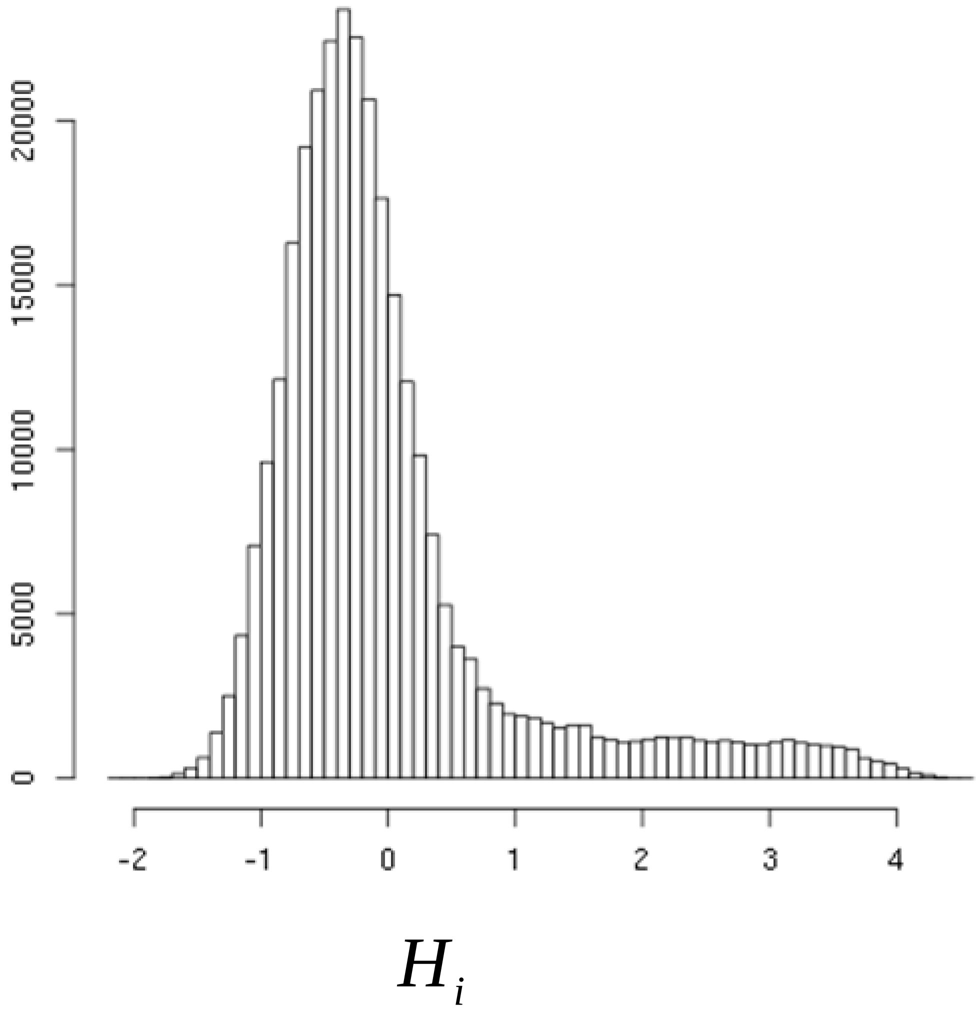
20000
15000
10000
5000
0
-2
-1
0
1
2
3
4
H_i
